# Supplementary material for: Machine learning-supported framework for the classification of mpox infection and MVA immunization from multiplexed serology data
Source: Nat Commun. 2025 Dec 5;16:10943. doi: 10.1038/s41467-025-66994-0 (PMC12686480; doi:10.1038/s41467-025-66994-0)
Supplement: Supplementary file 1 — Supplementary Information [file 41467_2025_66994_MOESM1_ESM.pdf]

Supplementary Information

Supplementary figures

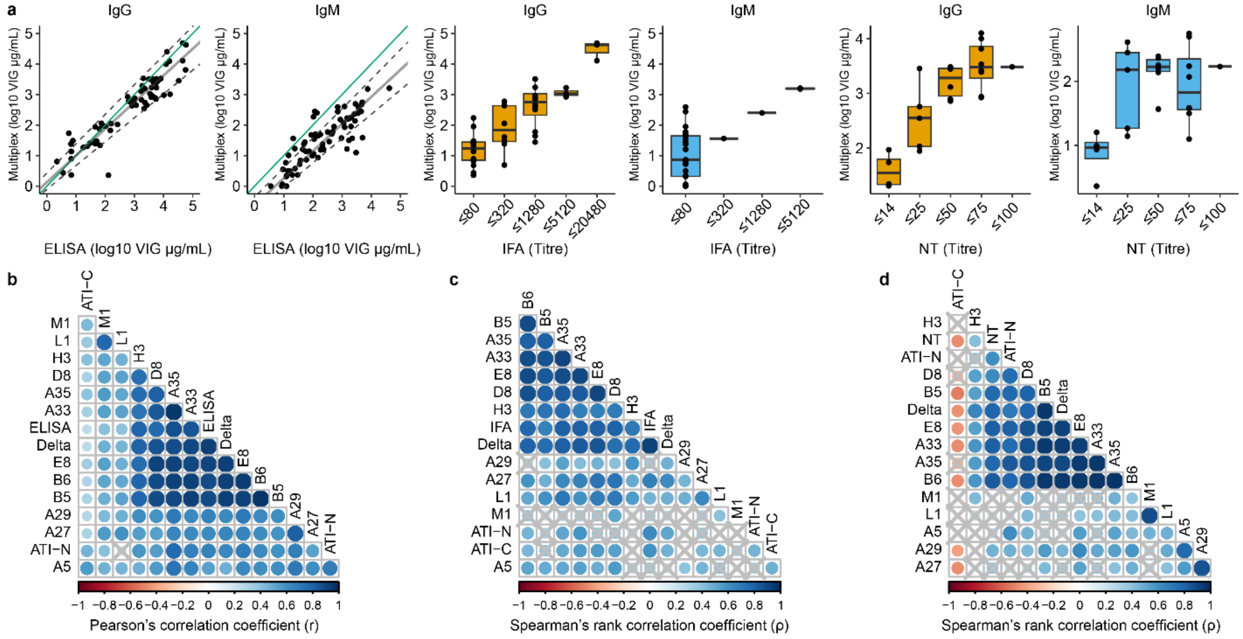

Supplementary Fig. 1. Comparison of multiplex assay results with ELISA, IFA, and neutralization test (NT) reference methods. a) Quantitative comparison of IgG (orange bars) and IgM (blue bars) responses to the Delta-VACV antigen across the multiplex assay and each of the three reference assays: ELISA (n = 75), IFA (n = 25), and NT (n = 25). Passing–Bablok regression shown: green line indicates identity, grey line shows fitted regression with 95% confidence intervals (dashed). b) Pearson correlation of multiplex IgG binding to individual recombinant antigens with ELISA results. c, d) Spearman correlation of IgG responses to recombinant antigens in the multiplex assay with IFA titres (c) and NT titres (d). Multiplex Delta quant./ELISA quant.: Units in ng/mL on a $\log_{10}$ scale as quantified against a standard curve of VIG. IFA, immunofluorescence assay; NT, neutralization test; VACV, vaccinia virus; VIG, vaccinia immune globulin. Source data are provided as Source Data file.

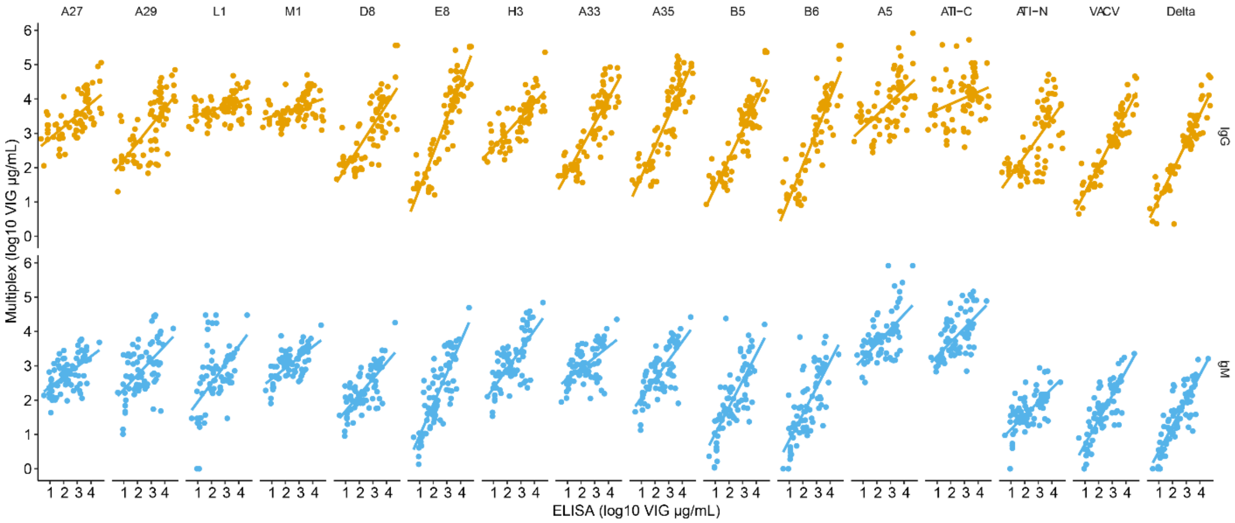

Supplementary Fig. 2. Comparison of IgG and IgM signals between an in-house ELISA and the orthopoxvirus-specific antigens in the multiplex assay. Quantified IgG (in orange) and IgM (in blue) levels (using a standard curve of VIG) were determined by ELISA and multiplex assay. ELISA Delta-VACV signals: binding to VACV antigen minus Hep-2 background versus antigen-specific results of the multiplex assay. Measurements were taken from distinct samples (n = 75). Multiplex quant./ELISA quant.: Units in µg/mL on a $\log_{10}$ scale as quantified against a standard curve of VIG. VIG, vaccinia immune globulin; VACV, vaccinia virus**.** Source data are provided as Source Data file.

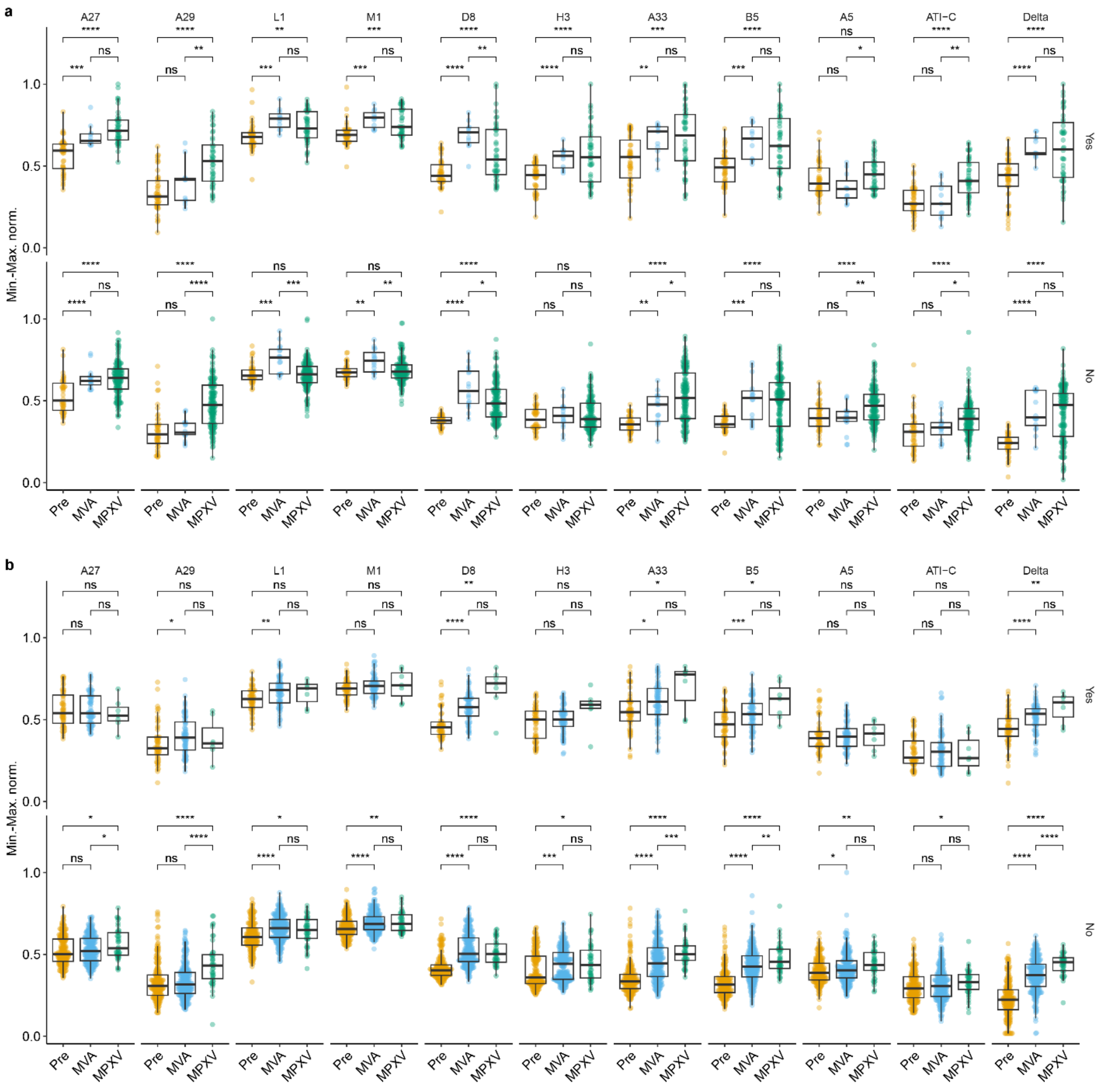

Supplementary Fig. 3. Box plots for pairwise comparisons of normalized IgG datasets for selected antigens in the acute (a) and epi dataset (b). Results are stratified by serum group (Pre, MVA, MPXV), antigen, and presumed childhood smallpox vaccinee (yes or no, sera from individuals between 40 and 49 years old were excluded). Statistically significant differences between serogroups (*t*-test, two-sided) indicated as asterisks (ns; * $p < 0.05$; ** $p < 0.01$; *** $p < 0.001$; **** $p < 0.0001$) based on the following number of sera in the respective groups: Acute cohort with and without childhood vaccination, 45 and 44 (Pre), 10 and 16 (MVA), 41 and 161 (MPXV). Epi cohort with/without childhood vaccination, 57 and 222 (Pre), 73 and 291 (MVA), 7 and 41 (MPXV). Measurements were taken from distinct samples. IgG (orange), IgM (blue); Pre, pre-immune; VACV, vaccinia virus; MPXV, mpox virus; MVA, modified vaccinia ankara; epi, epidemiological; ns, not significant. Source data are provided as a Source Data file. Exact p-values are provided in the Source Data file.

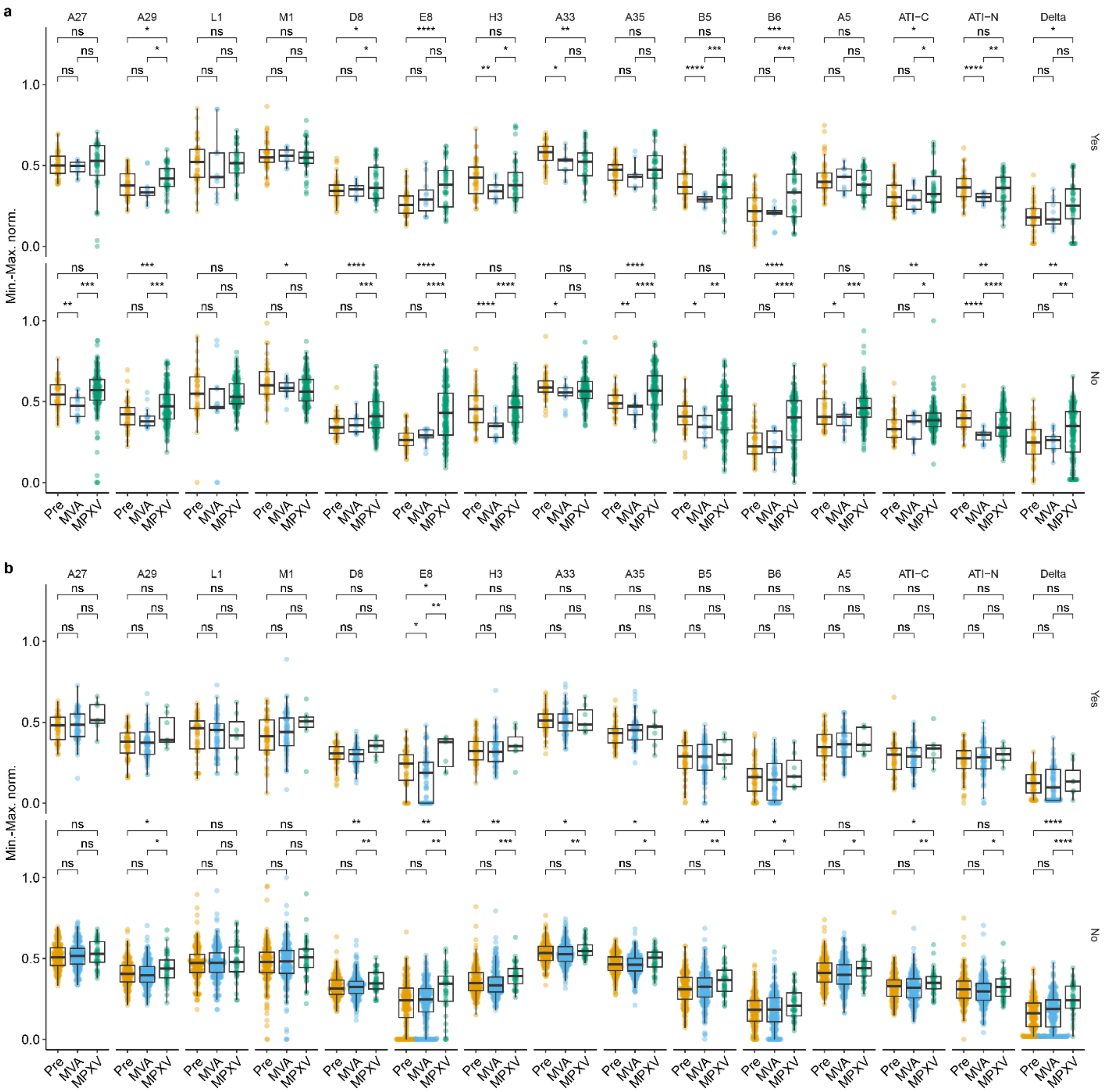

Supplementary Fig. 4. Box plots for pairwise comparisons of normalized IgM datasets for tested antigens in the acute (a) and the epi dataset (b). Results are stratified by serum group (Pre, MVA, MPXV), antigen, and presumed childhood smallpox vaccinee (sera from individuals between 40 and 49 years old were excluded). Statistically significant differences between serogroups (*t*-test, two-sided) indicated as asterisks (ns; * $p < 0.05$; ** $p < 0.01$; *** $p < 0.001$; **** $p < 0.0001$) based on the following number of sera in the respective groups: Acute cohort with and without childhood vaccination, 45 and 44 (Pre), 10 and 16 (MVA), 41 and 161 (MPXV). Epi cohort with and without childhood vaccination, 57 and 222 (Pre), 73 and 291 (MVA), 7 and 41 (MPXV). Measurements were taken from distinct samples. IgG (orange), IgM (blue); Pre, pre-immune; VACV, vaccinia virus, MPXV, mpox virus; MVA, modified vaccinia ankara; epi, epidemiological; ns, not significant. Source data are provided as a Source Data file. Exact p-values are provided in the Source Data file.

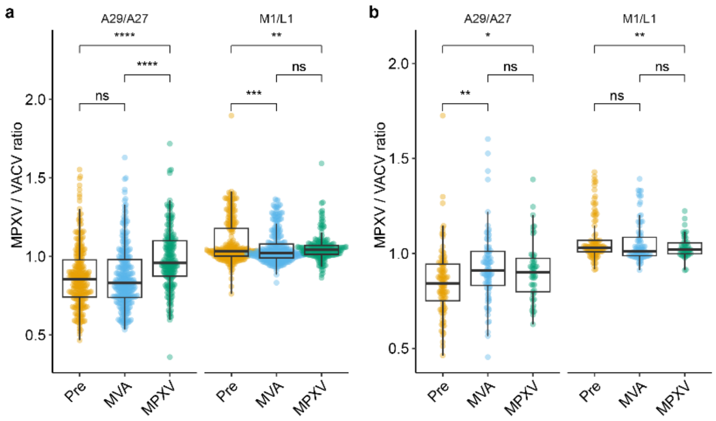

Supplementary Fig. 5. Box plots for pairwise comparison of selected IgG ratios in both cohorts (a: naïve, b: childhood vaccination) between MPXV and VACV homologous antigen pairs, respectively stratified by serum cohort (Pre, MVA, MPXV), antigen, and likely childhood smallpox vaccination (sera from individuals between 40 and 49 years old were excluded). Statistically significant differences between serogroups (*t*-test, two-sided) indicated as asterisks (ns; * $p < 0.05$; ** $p < 0.01$; *** $p < 0.001$; **** $p < 0.0001$) based on the following number of sera in the respective groups: Acute cohort with/without childhood vaccination, 45/44 (Pre), 10/16 (MVA), 41/161 (MPXV). Epi cohort with/without childhood vaccination, 57/222 (Pre), 73/291 (MVA), 7/41 (MPXV). Measurements were taken from distinct samples. Source data are provided as a Source Data file. Exact p-values are provided in the Source Data file.

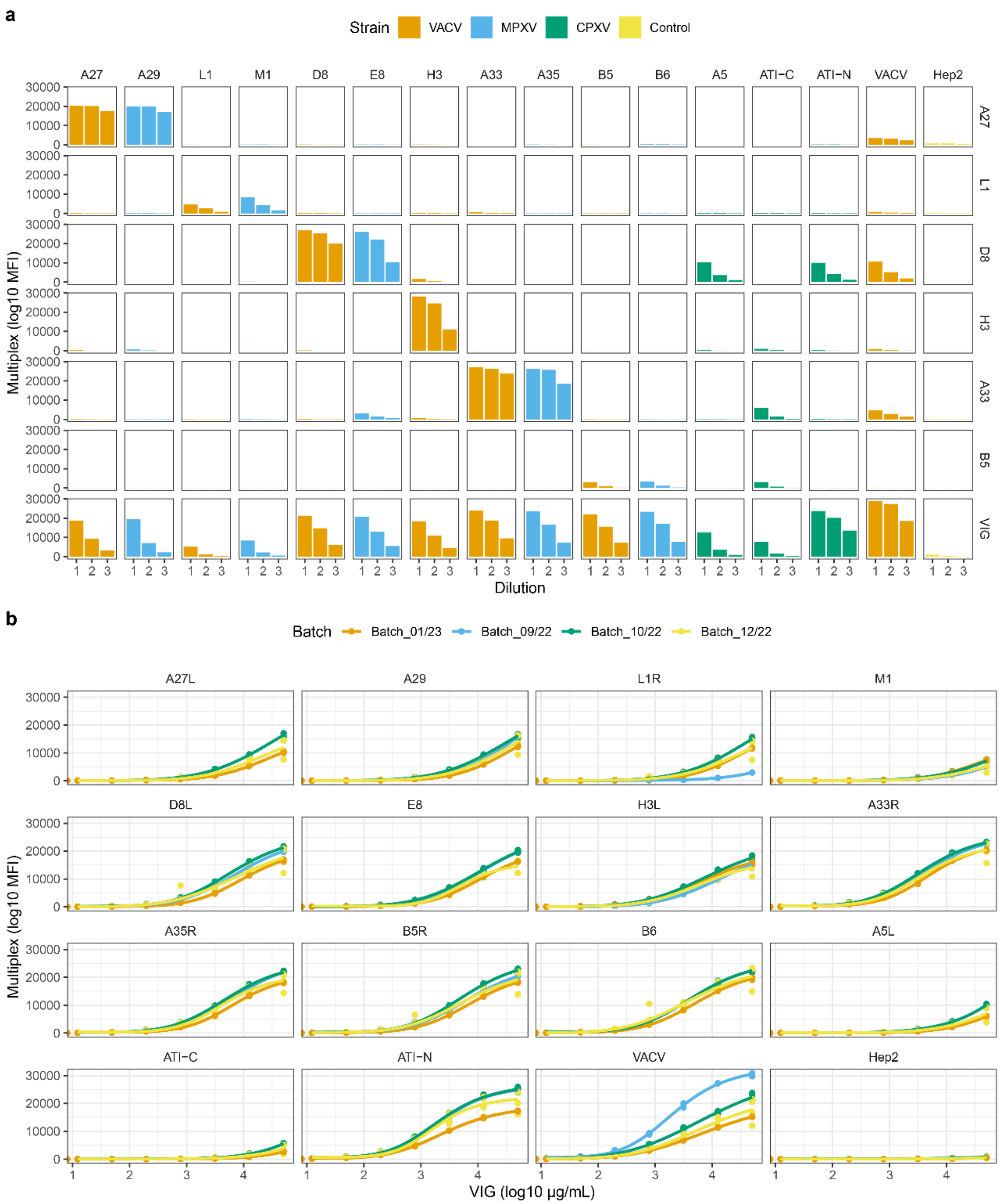

Supplementary Fig. 6. Binding of monospecific antibodies and VIG to *orthopoxvirus* specific antigens coupled to MagPlex beads and analysis of batch to batch variability of coupling. a) Test of three different dilutions of mono- or polyclonal monospecific antibodies and VIG (vertical facets) targeting antigens coupled to MagPlex beads (horizontal facets). b) Binding of a 1:4 serial dilution of VIG (from 10,000 µg/mL to 39 µg/mL) to orthopoxvirus-specific antigens conjugated to four separate bead batches. VIG, vaccinia immune globulin; VACV, vaccinia virus, MPXV, mpox virus; CPXV, cowpox virus. Source data are provided as a Source Data file.

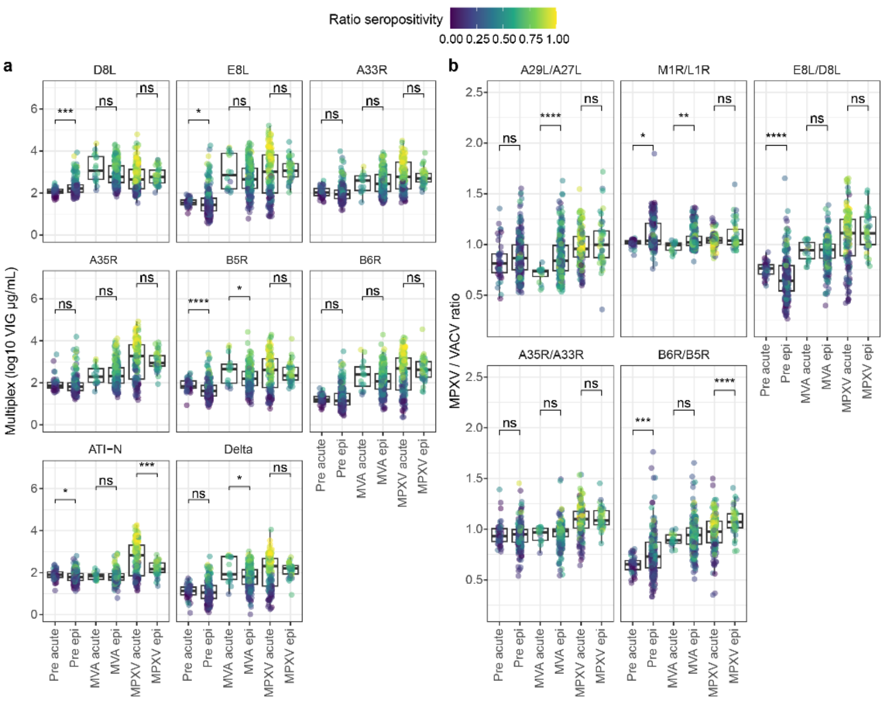

Supplementary Fig. 7. Binding profiles of IgG antibodies to a panel of highly immunogenic antigens. a) Pairwise comparison of IgG binding to selected immunodominant antigens using sera from individuals <40 years old across three serum status groups (Pre, MVA, and MPXV) in both tested serological cohorts (epi and acute). b) Corresponding comparisons of binding ratios between homologous VACV/MPXV antigen pairs. Statistical significance was determined using two-sided Student's t-tests (ns; *$p < 0.05$; **$p < 0.01$; ***$p < 0.001$; ****$p < 0.0001$). Population-based cut-off values for exclusion from ML training/testing were defined via ROC analysis. For most antigens, pre-immune sera from the acute cohort were used as the negative group and MPXV-infected sera from the epidemiological cohort as the positive group. For ATI-N-CPXV, both pre-immune and MVA-vaccinated sera from the acute cohort were included as the negative group. ROC curves were also generated for E8-MPXV/D8-VACV, A35-MPXV/A33-VACV, and B6-MPXV/B5-VACV ratios, with MVA-vaccinated sera (pre-immune group) as the negative and MPXV-infected sera as the positive group. Corresponding ROC curves are shown in Fig. S8. Colour coding reflects the serostatus (fraction seropositive) across orthopoxvirus-specific antigens. Measurements were performed on distinct biological samples. Pre, pre-immune; MVA, modified vaccinia ankara; MPXV, mpox virus; epi, epidemiological; ns, not significant; ROC, Receiver Operating Characteristic; CPXV, cowpox virus. Source data are provided as a Source Data file.

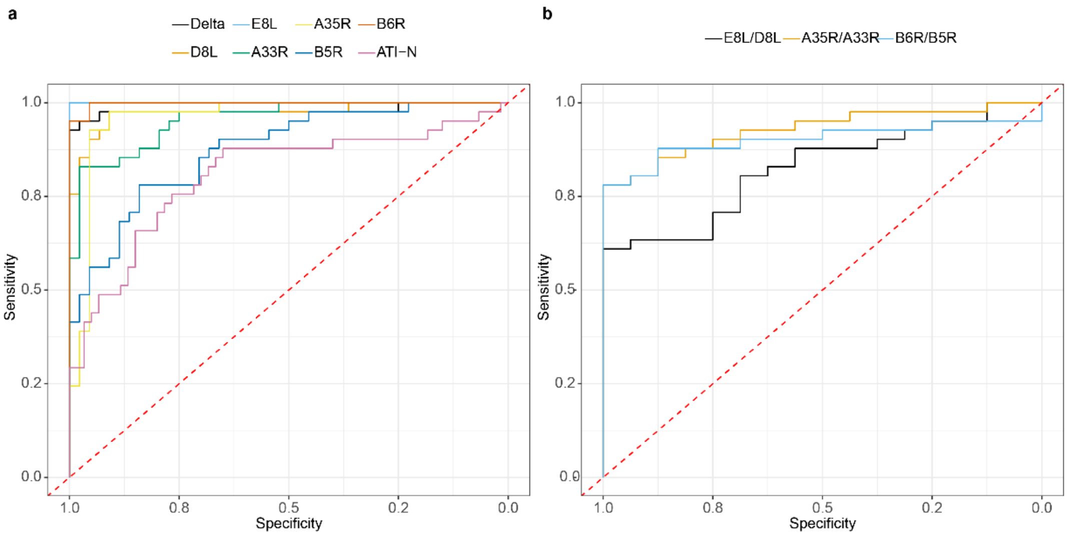

Supplementary Fig. 8. ROC curves used to define population-based cut-off values for excluding sera from ML model training and testing, based on antigen-specific IgG binding. a) ROC curves for individual antigens. b) ROC curves for binding ratios between homologous MPXV/VACV antigen pairs, as detailed in Fig. S7. Sera from the epi cohort were excluded from ML training datasets based on the following criteria: Pre sera (n = 95): exclusion required at least one antigen exceeding a cut-off value set to achieve 100% specificity, or at least two antigen ratios exceeding their respective cut-off values. MVA-vaccinated sera (n = 37): exclusion required ATI-N-CPXV binding above the 100% specificity cut-off, or at least two ratios above their respective cut-offs plus at least one additional antigen above its 100% specificity threshold. These thresholds were determined using ROC analysis and represent population-level standards for ensuring high specificity in training data. ROC, Receiver Operating Characteristic; ML, machine learning; MPXV, mpox virus; VACV, vaccinia virus, epi, epidemiological; pre, pre-immune; MVA, modified vaccinia ankara; CPXV, cowpox virus. Source data are provided as a Source Data file.

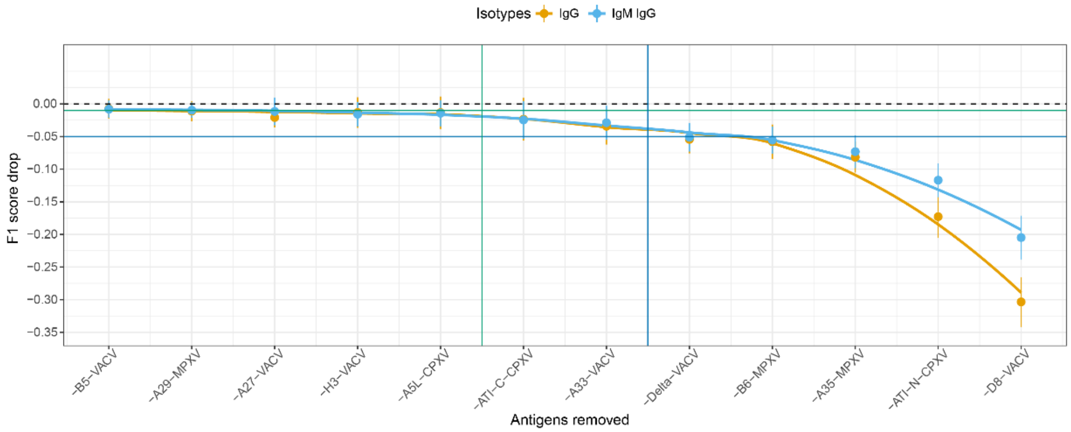

Supplementary Fig 9. Recursive feature elimination assessing the impact of cumulatively removing the least informative antigens on the performance of the GBC model. Macro F1 score drop (mean ±SD) from 15 cross-validation runs. Horizontal lines indicate baseline performance (dashed line F1 score decreases of 0.01 (green) or 0.05 (blue)); corresponding vertical lines indicate the number or identity of antigens whose removal reduces the mean F1 score below these thresholds. GBC, Gradient Boosting Classifier; VACV, vaccinia virus, MPXV, mpox virus; CPXV, cowpox virus. Source data are provided as a Source Data file.

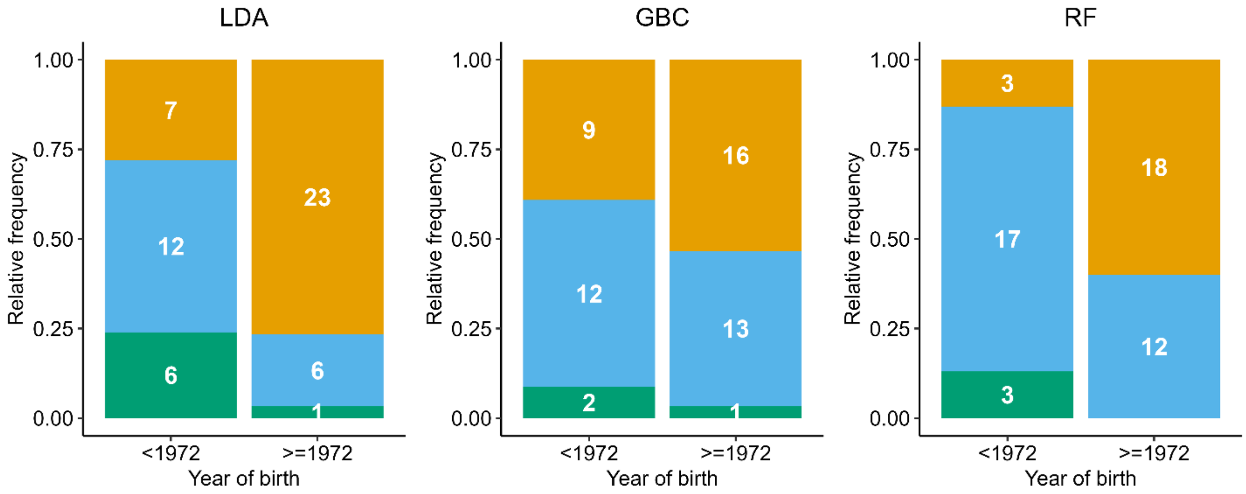

Supplementary Fig. 10. Misclassifications in the pre-immune group, stratified by year of birth. Classification in the independent validation panel, stratified by year of birth as proxy for childhood smallpox immunization. Orange: classified as pre-immune (correct). Blue: classified as MVA (misclassified). Green: classified as MPXV-infected (misclassified). Misclassifications are more frequent in sera with presumed childhood smallpox vaccination. Pre-immune sera are more often misclassified as MVA-vaccinated. LDA shows fewer misclassifications in the pre-immune group, followed by RF and GBC. MVA, modified vaccinia ankara; MPXV, mpox virus; LDA, Linear Discriminant Analysis; RF, Random Forest; GBC, Gradient Boosting Classifier. Source data are provided as a Source Data file.

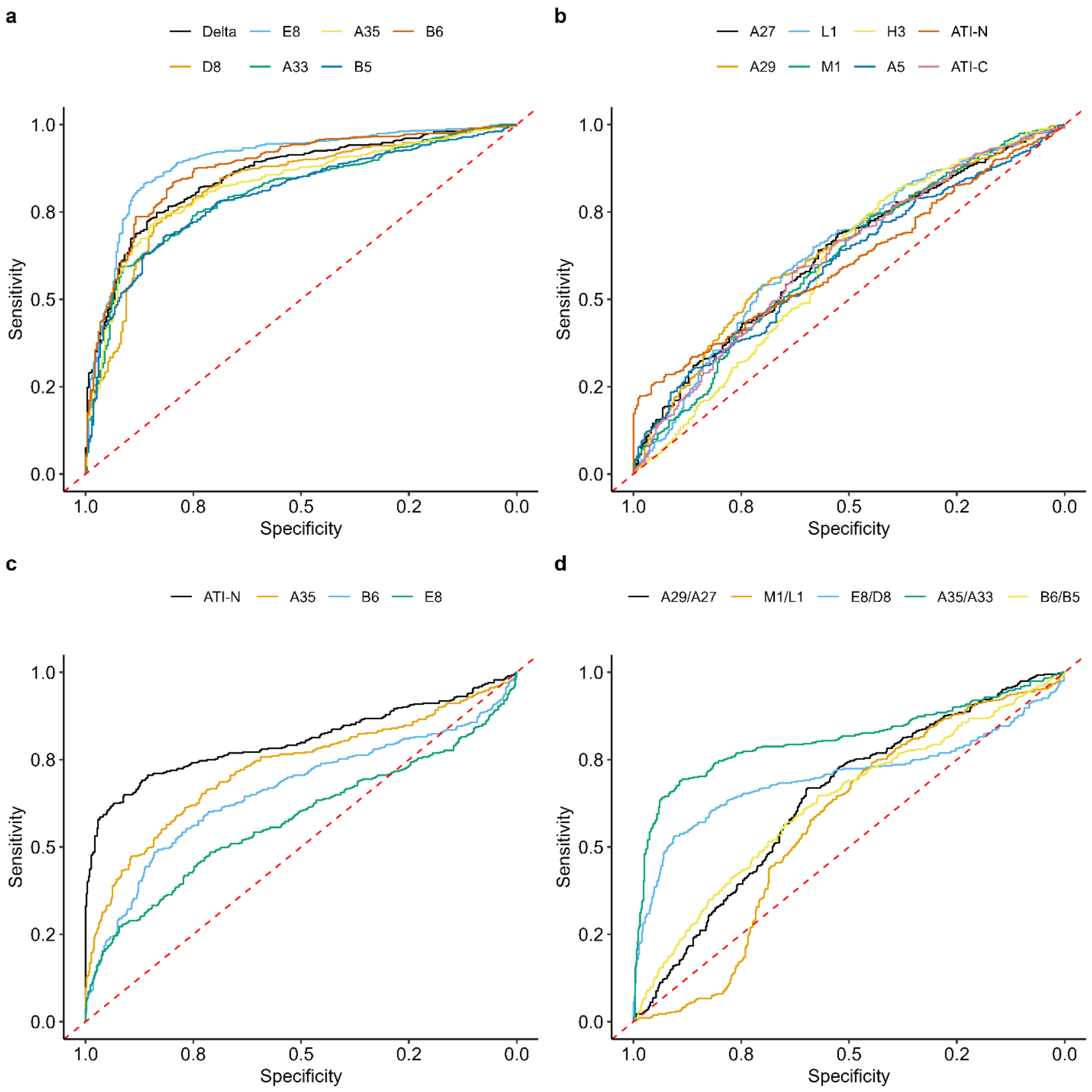

Supplementary Fig. 11. ROC curves to distinguish MPXV infection and MVA vaccination based on single antigens and impact of filtering based on ML-intrinsic metrics on prediction accuracy. Sensitivity and specificity to distinguish between pre-immune sera as orthopoxvirus seronegative (n = 206) or seropositive, either post MVA-vaccinated (n = 307) or post MPXV-infected (n = 202) combined from both the acute and epi cohort in subjects younger than 40 years based on single antigens with good (a) or poor (b) performance. c) Differentiation between MVA-vaccinated (n = 512) or MPXV-infected (n = 363) from both the acute and epi cohort based on selected antigens across all age groups. d) Differentiation between MVA-vaccinated (n = 512) or MPXV-infected (n = 363) from both the acute and epi cohort based on ratio of binding to MPXV versus VACV homologous antigens across all age groups. Metrics describing the ROC performance characteristics are shown in Supplementary Table 12. ROC, Receiver Operating Characteristic; MPXV, mpox virus; MVA, modified vaccinia ankara; ML, machine learning; epi, epidemiological; VACV, vaccinia virus. Source data are provided as a Source Data file.

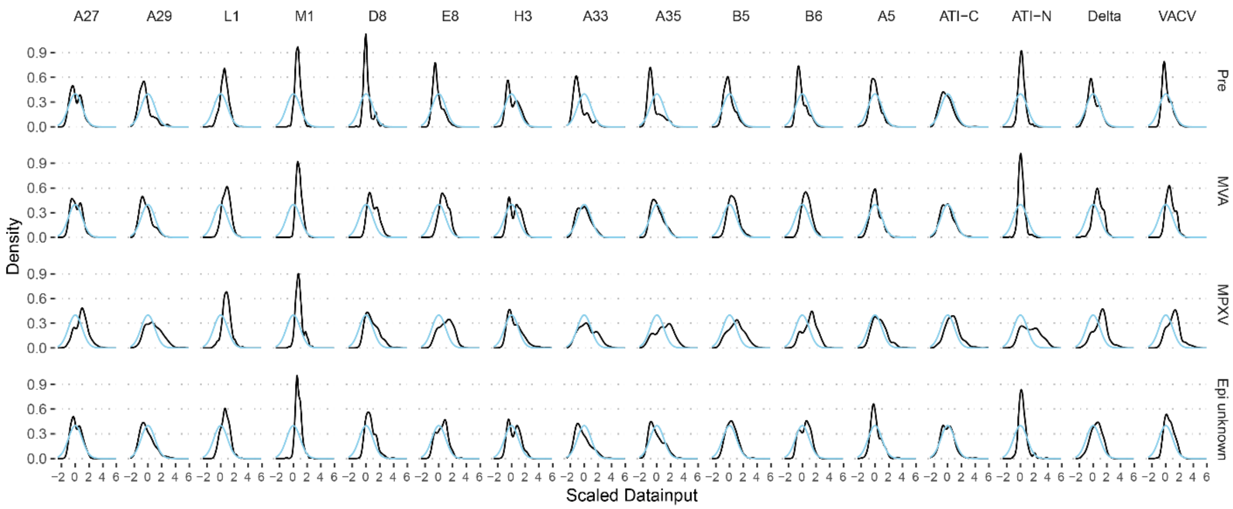

Supplementary Fig. 12. Density plots of scaled IgG data stratified by antigen and serum group. Scaled IgG values (mean = 0, SD = 1) for all 1,569 samples are shown as empirical density (black) overlaid with the standard normal distribution (blue), stratified by antigen and serum group (Pre, MVA, MPXV, and Epi unknown, containing samples from the epidemiological cohort without metadata, hence not attributable to one of the three serum groups). Deviations from normality are evident and confirmed by Shapiro–Wilk tests for all groups. SPox: samples from the epidemiological cohort without metadata, hence not attributable to one of the three serum groups. SD, standard deviation. Source data are provided as a Source Data file.

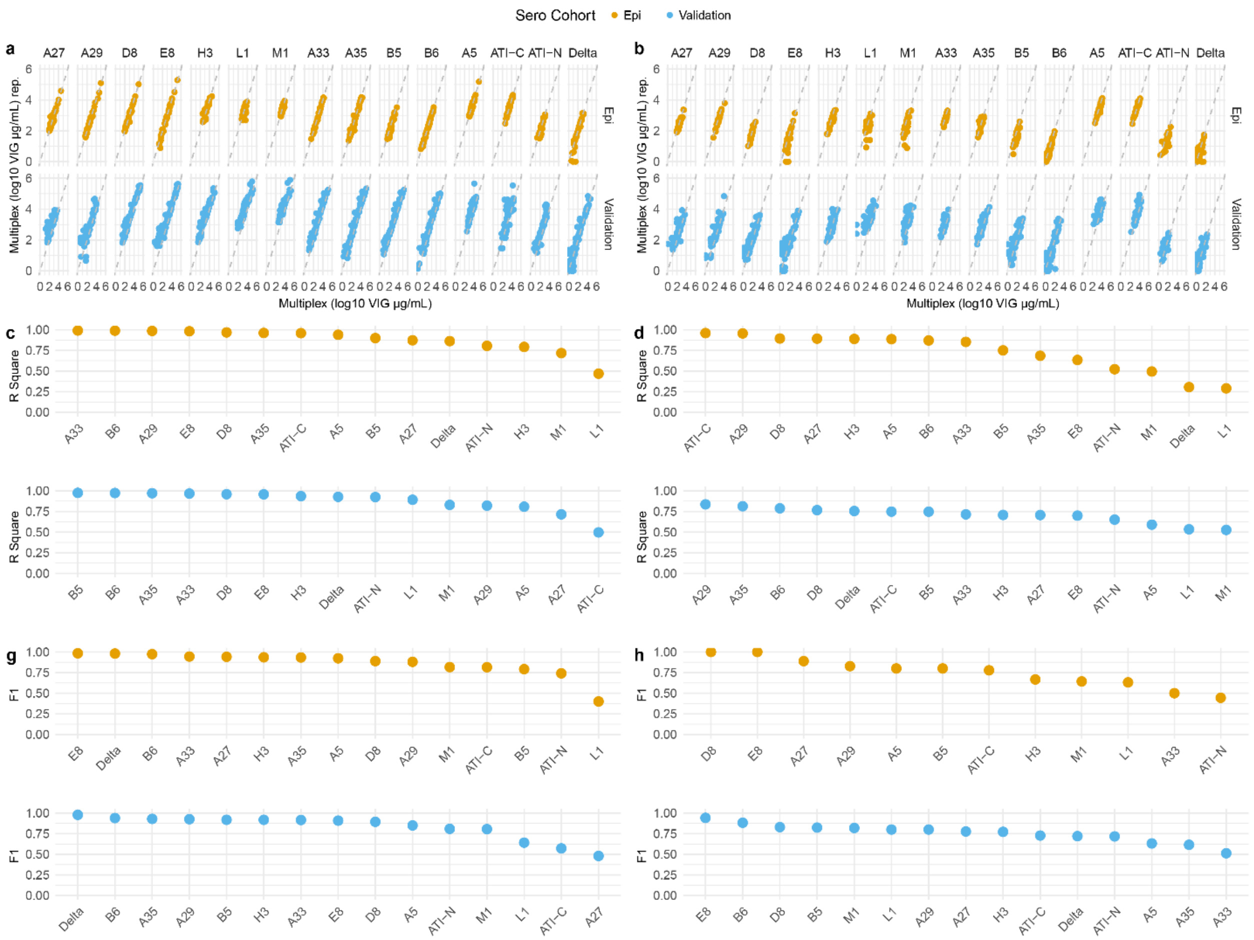

Supplementary Fig. 13. Reproducibility of IgG and IgM measurements across independent assays. (a, b) Scatter plots comparing quantified IgG (a) and IgM (b) levels across two independent measurements, stratified by antigen and serological cohort (epi, n = 39; validation, n = 139). Each point represents a single serum sample. (c, d) Coefficient of determination (R^2) for linear regression between the two measurements, shown separately for the epidemiological (orange) and validation (blue) cohorts, for IgG (c) and IgM (d). (g, h) F1 scores quantifying agreement in binary classification as seropositive or seronegative for each antigen, based on cut-off values established by method comparison with IFA (ground truth: titres 1:320 or above seropositive), for IgG (g) and IgM (h). The measurements on the validation cohort were performed by two operators using two batches of coupled beads on two different instruments while the measurements on the epi cohort were performed by one operator using the same batch of beads. IFA, immunofluorescence assay; epi, epidemiological. Source data are provided as a Source Data file.

Supplementary tables

Supplementary Table 1. Pairwise sequence homology between the ATI protein from different *Orthopoxvirus* strains[#] based on the amino acid sequences.

ATI-CPXV	MPXV_Zaire	CPXV_BR	VACV_Dryvax	VACV_Acambis	VACV_WR	Variola_major	Variola_minor
MPXV_Zaire		91.7	94.4	94.4	94.8	93.1	93.0
CPXV_BR	91.7		92.1	92.1	92.5	87.5	87.4
VACV_Dryvax	94.4	92.1		100.0	98.8	90.9	90.8
VACV_Acambis	94.4	92.1	100.0		98.8	90.9	90.8
VACV_WR	94.8	92.5	98.8	98.8		91.6	91.5
Variola_major	93.1	87.5	90.9	90.9	91.6		99.9
Variola_minor	93.0	87.4	90.8	90.8	91.5	99.9	

[#] BR: Brighton Red, WR: Western Reserve. MPXV, mpox virus; CPXV, cowpox virus; VACV, vaccinia virus.

Supplementary Table 2. Pairwise sequence homology between the A5-CPXV protein from different *Orthopoxvirus* strains[#] based on the amino acid sequences.

A5-CPXV	MPXV_Zaire	CPXV_BR	VACV_MVA	VACV_Dryvax	VACV_Acambis	VACV_WR	Variola_major	Variola_minor
MPXV_Zaire		87.1	91.8	92.6	92.6	94.0	85.0	84.6
CPXV_BR	87.1		85.8	87.5	87.5	86.8	83.4	83.8
VACV_MVA	91.8	85.8		93.6	93.6	95.4	89.9	90.3
VACV_Dryvax	92.6	87.5	93.6		100.0	95.8	86.4	86.7
VACV_Acambis	92.6	87.5	93.6	100.0		95.8	86.4	86.7
VACV_WR	94.0	86.8	95.4	95.8	95.8		85.7	86.0
Variola_major	85.0	83.4	89.9	86.4	86.4	85.7		98.9
Variola_minor	84.6	83.8	90.3	86.7	86.7	86.0	98.9	

[#] BR: Brighton Red, WR: Western Reserve. MPXV, mpox virus; CPXV, cowpox virus; VACV, vaccinia virus.

Supplementary Table 3. Pairwise sequence homology between the A33-VACV/A35-MPXV proteins from different *Orthopoxvirus* strains[#] based on the amino acid sequences.

A33-VACV/A35-MPXV	MPXV_Zaire	CPXV_BR	VACV_MVA	VACV_Dryvax	VACV_Acambis	VACV_WR	Variola_major	Variola_minor
MPXV_Zaire		92.3	95.0	95.6	95.6	95.6	88.5	88.5
CPXV_BR	92.3		93.0	93.6	93.6	93.6	88.8	88.8
VACV_MVA	95.0	93.0		98.4	98.4	98.4	90.3	90.3
VACV_Dryvax	95.6	93.6	98.4		100.0	100.0	90.9	90.9
VACV_Acambis	95.6	93.6	98.4	100.0		100.0	90.9	90.9
VACV_WR	95.6	93.6	98.4	100.0	100.0		90.9	90.9
Variola_major	88.5	88.8	90.3	90.9	90.9	90.9		100.0
Variola_minor	88.5	88.8	90.3	90.9	90.9	90.9	100.0	

[#] BR: Brighton Red, WR: Western Reserve. MPXV, mpox virus; CPXV, cowpox virus; VACV, vaccinia virus.

Supplementary Table 4. Pairwise sequence homology between the B5-VACV/B6-MPXV proteins from different *Orthopoxvirus* strains# based on the amino acid sequences.

B5-VACV/B6-MPXV	MPXV_Zaire	CPXV_BR	VACV_MVA	VACV_Dryvax	VACV _Acambis	VACV_WR	Variola_major	Variola_minor
MPXV_Zaire		93.7	96.2	96.5	96.8	96.5	92.4	92.7
CPXV_BR	93.7		92.7	93.1	93.4	93.4	93.7	94.0
VACV_MVA	96.2	92.7		99.7	99.4	97.2	92.4	92.7
VACV_Dryvax	96.5	93.1	99.7		99.7	96.8	92.7	93.1
VACV_Acambis	96.8	93.4	99.4	99.7		96.5	93.1	93.4
VACV_WR	96.5	93.4	97.2	96.8	96.5		92.7	93.1
Variola_major	92.4	93.7	92.4	92.7	93.1	92.7		99.7
Variola_minor	92.7	94.0	92.7	93.1	93.4	93.1	99.7	

BR: Brighton Red, WR: Western Reserve. MPXV, mpox virus; CPXV, cowpox virus; VACV, vaccinia virus.

Supplementary Table 5. Pairwise sequence homology between the A27-VACV/A29-MPXV proteins from different *Orthopoxvirus* strains# based on the amino acid sequences.

A27-VACV/A29-MPXV	MPXV_Zaire	CPXV_BR	VACV_MVA	VACV_Dryvax	VACV_Acambis	VACV_WR	Variola_major	Variola_minor
MPXV_Zaire		95.5	93.6	94.5	94.5	94.5	94.5	94.5
CPXV_BR	95.5		97.3	98.2	98.2	98.2	98.2	98.2
VACV_MVA	93.6	97.3		99.1	99.1	99.1	97.3	97.3
VACV_Dryvax	94.5	98.2	99.1		100.0	100.0	98.2	98.2
VACV_Acambis	94.5	98.2	99.1	100.0		100.0	98.2	98.2
VACV_WR	94.5	98.2	99.1	100.0	100.0		98.2	98.2
Variola_major	94.5	98.2	97.3	98.2	98.2	98.2		100.0
Variola_minor	94.5	98.2	97.3	98.2	98.2	98.2	100.0	

BR: Brighton Red, WR: Western Reserve. MPXV, mpox virus; CPXV, cowpox virus; VACV, vaccinia virus.

Supplementary Table 6. Pairwise sequence homology between the D8-VACV/E8-MPXV proteins from different *Orthopoxvirus* strains# based on the amino acid sequences.

D8-VACV/E8-MPXV	MPXV_Zaire	CPXV_BR	VACV_MVA	VACV_Dryvax	VACV_Acambis	VACV_WR	Variola_major	Variola_minor
MPXV_Zaire		97.0	94.4	94.1	94.1	94.1	92.8	92.8
CPXV_BR	97.0		94.1	94.4	94.4	94.4	93.4	93.4
VACV_MVA	94.4	94.1		98.4	97.7	98.4	95.7	95.7
VACV_Dryvax	94.1	94.4	98.4		99.3	99.3	96.7	96.7
VACV_Acambis	94.1	94.4	97.7	99.3		98.7	97.4	97.4
VACV_WR	94.1	94.4	98.4	99.3	98.7		96.1	96.1
Variola_major	92.8	93.4	95.7	96.7	97.4	96.1		100.0
Variola_minor	92.8	93.4	95.7	96.7	97.4	96.1	100.0	

BR: Brighton Red, WR: Western Reserve. MPXV, mpox virus; CPXV, cowpox virus; VACV, vaccinia virus.

Supplementary Table 7. Pairwise sequence homology between the H3-VACV proteins from different *Orthopoxvirus* strains[#] based on the amino acid sequences.

H3-VACV	MPXV_Zaire	CPXV_BR	VACV_MVA	VACV_Dryvax	VACV_Acambis	VACV_WR	Variola_major	Variola_minor
MPXV_Zaire		94.1	93.8	92.6	94.1	93.8	93.2	93.5
CPXV_BR	94.1		96.6	94.8	96.3	95.7	95.1	95.4
VACV_MVA	93.8	96.6		97.5	99.7	98.8	96.0	96.3
VACV_Dryvax	92.6	94.8	97.5		97.8	96.9	94.4	94.8
VACV_Acambis	94.1	96.3	99.7	97.8		99.1	96.3	96.6
VACV_WR	93.8	95.7	98.8	96.9	99.1		96.0	96.3
Variola_major	93.2	95.1	96.0	94.4	96.3	96.0		99.7
Variola_minor	93.5	95.4	96.3	94.8	96.6	96.3	99.7	

[#] BR: Brighton Red, WR: Western Reserve. MPXV, mpox virus; CPXV, cowpox virus; VACV, vaccinia virus.

Supplementary Table 8. Pairwise sequence homology between the L1-VACV/M1- MPXV proteins from different *Orthopoxvirus* strains[#] based on the amino acid sequences.

L1-VACV/M1-MPXV	MPXV_Zaire	CPXV_BR	VACV_MVA	VACV_Dryvax	VACV_Acambis	VACV_WR	Variola_major	Variola_minor
MPXV_Zaire		98.4	98.8	98.8	98.8	98.4	99.2	98.8
CPXV_BR	98.4		98.8	98.8	98.8	98.4	99.2	98.8
VACV_MVA	98.8	98.8		100.0	100.0	99.6	99.6	99.2
VACV_Dryvax	98.8	98.8	100.0		100.0	99.6	99.6	99.2
VACV_Acambis	98.8	98.8	100.0	100.0		99.6	99.6	99.2
VACV_WR	98.4	98.4	99.6	99.6	99.6		99.2	98.8
Variola_major	99.2	99.2	99.6	99.6	99.6	99.2		99.6
Variola_minor	98.8	98.8	99.2	99.2	99.2	98.8	99.6	

[#] BR: Brighton Red, WR: Western Reserve. MPXV, mpox virus; CPXV, cowpox virus; VACV, vaccinia virus.

Supplementary Table 9. Performance parameters for tested ML-algorithms stratified by cohort used for training and testing and antibody isotypes. Shown are mean values ± standard deviation of 15 models. Source data are provided as a Source Data file.

Serological cohort[#]	Isotypes	Parameter	LDA	RF	GBC	FRBC	LDA+FRBC	LDA+RF
acute acute	IgG	accuracy	0.81 ±0.03	0.83 ±0.02	0.86 ±0.03	0.77 ±0.02	0.76 ±0.03	0.8 ±0.03
acute acute	IgG	precision	0.79 ±0.05	0.85 ±0.05	0.84 ±0.04	0.75 ±0.06	0.51 ±0.07	0.79 ±0.05
acute acute	IgG	recall	0.77 ±0.06	0.73 ±0.03	0.81 ±0.05	0.7 ±0.06	0.54 ±0.03	0.76 ±0.06
acute acute	IgG	F1 score	0.78 ±0.05	0.76 ±0.04	0.82 ±0.04	0.71 ±0.04	0.52 ±0.04	0.77 ±0.05
acute acute	IgM IgG	accuracy	0.87 ±0.03	0.85 ±0.03	0.87 ±0.02	0.83 ±0.03	0.83 ±0.02	0.87 ±0.03
acute acute	IgM IgG	precision	0.83 ±0.06	0.88 ±0.03	0.85 ±0.05	0.78 ±0.09	0.56 ±0.02	0.85 ±0.05
acute acute	IgM IgG	recall	0.81 ±0.07	0.74 ±0.06	0.81 ±0.05	0.75 ±0.07	0.59 ±0.02	0.81 ±0.05
acute acute	IgM IgG	F1 score	0.82 ±0.06	0.78 ±0.06	0.82 ±0.04	0.76 ±0.07	0.57 ±0.02	0.82 ±0.05
acute epi	IgG	accuracy	0.55 ±0	0.37 ±0	0.38 ±0.01	0.42 ±0.06	0.33 ±0	0.56 ±0
acute epi	IgG	precision	0.56 ±0	0.51 ±0	0.49 ±0	0.49 ±0.02	0.21 ±0	0.57 ±0
acute epi	IgG	recall	0.64 ±0	0.54 ±0	0.49 ±0.01	0.47 ±0.04	0.51 ±0	0.65 ±0
acute epi	IgG	F1 score	0.51 ±0	0.34 ±0	0.36 ±0.01	0.39 ±0.05	0.3 ±0	0.52 ±0
acute epi	IgM IgG	accuracy	0.51 ±0	0.31 ±0	0.4 ±0	0.36 ±0.03	0.23 ±NA	0.46 ±0.01
acute epi	IgM IgG	precision	0.52 ±0	0.48 ±0	0.48 ±0	0.46 ±0.02	0.16 ±NA	0.5 ±0
acute epi	IgM IgG	recall	0.54 ±0	0.46 ±0.01	0.49 ±0	0.42 ±0.03	0.43 ±NA	0.52 ±0.01
acute epi	IgM IgG	F1 score	0.46 ±0	0.29 ±0	0.38 ±0	0.33 ±0.03	0.21 ±NA	0.43 ±0
all all	IgG	accuracy	0.78 ±0.02	0.78 ±0.02	0.81 ±0.02	0.76 ±0.02	0.63 ±0.02	0.79 ±0.02
all all	IgG	precision	0.8 ±0.02	0.79 ±0.02	0.81 ±0.02	0.76 ±0.02	0.66 ±0.02	0.79 ±0.02
all all	IgG	recall	0.78 ±0.02	0.76 ±0.02	0.81 ±0.02	0.75 ±0.03	0.64 ±0.02	0.78 ±0.02
all all	IgG	F1 score	0.78 ±0.02	0.77 ±0.02	0.81 ±0.02	0.75 ±0.03	0.64 ±0.02	0.78 ±0.02
all all	IgM IgG	accuracy	0.8 ±0.02	0.77 ±0.02	0.83 ±0.02	0.76 ±0.02	0.6 ±0.03	0.8 ±0.02
all all	IgM IgG	precision	0.81 ±0.02	0.8 ±0.02	0.83 ±0.02	0.76 ±0.02	0.61 ±0.06	0.81 ±0.02
all all	IgM IgG	recall	0.79 ±0.02	0.76 ±0.02	0.82 ±0.02	0.75 ±0.02	0.61 ±0.03	0.8 ±0.02
all all	IgM IgG	F1 score	0.8 ±0.02	0.76 ±0.03	0.82 ±0.02	0.75 ±0.02	0.6 ±0.04	0.8 ±0.02
epi acute	IgG	accuracy	0.67 ±0	0.46 ±0.01	0.61 ±0.01	0.5 ±0.07	0.43 ±0.01	0.67 ±0
epi acute	IgG	precision	0.62 ±0	0.6 ±0	0.59 ±0.01	0.56 ±0.03	0.59 ±0	0.62 ±0
epi acute	IgG	recall	0.76 ±0	0.64 ±0.01	0.71 ±0.01	0.65 ±0.04	0.59 ±0.01	0.76 ±0
epi acute	IgG	F1 score	0.62 ±0	0.45 ±0.01	0.56 ±0.01	0.47 ±0.06	0.42 ±0.01	0.62 ±0
epi acute	IgM IgG	accuracy	0.69 ±0	0.63 ±0	0.63 ±0.01	0.48 ±0.08	0.49 ±0.03	0.68 ±0

Serological cohort#	Isotypes	Parameter	LDA	RF	GBC	FRBC	LDA+FRBC	LDA+RF
epi acute	IgM IgG	precision	0.63 ±0	0.64 ±0	0.6 ±0.01	0.53 ±0.05	0.58 ±0.01	0.62 ±0
epi acute	IgM IgG	recall	0.76 ±0	0.73 ±0	0.71 ±0.01	0.62 ±0.05	0.62 ±0.01	0.76 ±0
epi acute	IgM IgG	F1 score	0.63 ±0	0.58 ±0	0.58 ±0.01	0.45 ±0.07	0.46 ±0.02	0.62 ±0
epi epi	IgG	accuracy	0.81 ±0.03	0.81 ±0.02	0.82 ±0.02	0.79 ±0.03	0.76 ±0.03	0.81 ±0.03
epi epi	IgG	precision	0.78 ±0.04	0.77 ±0.09	0.79 ±0.06	0.71 ±0.04	0.68 ±0.1	0.79 ±0.06
epi epi	IgG	recall	0.75 ±0.05	0.62 ±0.04	0.73 ±0.04	0.65 ±0.04	0.58 ±0.04	0.75 ±0.06
epi epi	IgG	F1 score	0.76 ±0.04	0.64 ±0.05	0.75 ±0.04	0.67 ±0.04	0.59 ±0.05	0.76 ±0.05
epi epi	IgM IgG	accuracy	0.82 ±0.03	0.8 ±0.02	0.83 ±0.02	0.78 ±0.03	0.77 ±0.03	0.81 ±0.02
epi epi	IgM IgG	precision	0.81 ±0.04	0.76 ±0.14	0.78 ±0.07	0.7 ±0.06	0.71 ±0.08	0.8 ±0.04
epi epi	IgM IgG	recall	0.77 ±0.04	0.59 ±0.03	0.71 ±0.05	0.64 ±0.04	0.62 ±0.06	0.74 ±0.05
epi epi	IgM IgG	F1 score	0.78 ±0.04	0.61 ±0.05	0.74 ±0.06	0.65 ±0.04	0.64 ±0.06	0.76 ±0.04

Serological cohort used for training and testing, e.g. acute acute: model trained on acute dataset, model tested on acute dataset. All all: trained and tested on the combined acute and epi dataset. ML, machine learning; LDA, Linear Discriminant Analysis; RF, Random Forest; GBC, Gradient Boosting Classifier; FRBC, Fuzzy Rule-based Classification.

Supplementary Table 10. Results for predictions performed in the validation panel (n = 143) using the ensemble learning algorithms. 'Real' represents the ground truth used to calculate the assay performance. The ensemble serostatus, or mean outcome from the ensemble prediction, was determined. This was based on either the Delta-VACV serostatus, the mean of the LDA and GBC algorithms, or the serostatus derived from the LDA, GBC, or RF algorithm alone. Delta-VACV serostatus was determined using cut-off values established by method comparison with IFA data (Supplementary Fig. 1), with titres < 1:320 considered negative. ATI-N represents the serostatus with regards to ATI-N-CPXV as a marker for recent infection, based on a population-based cut-off, determined as described in Supplementary Fig. 7. Additional metadata provided is the number of MVA vaccinations received, the year of birth, the confidence for predictions based on the ensemble or the LDA algorithm. Source data are provided as a Source Data file.

Sample	Real	LDA	GBC	RF	Ensemble mean	Ensemble sero	Serostatus (Delta)	Serostatus ATI-N-CPXV	MVA vacc. (n)	Year Birth	Ensemble conf. serostatus	Ensemble conf. mean	LDA conf.
1	MPXV	MVA	MVA	MVA	MVA	MVA	positive	negative	2	1989	1	1	0.86
2	MPXV	MPXV	MPXV	MVA	MPXV	MPXV	negative	positive			0.48	0.9	0.46
3	MPXV	MPXV	MPXV	MPXV	MPXV	MPXV	borderline positive	positive	0	1998	0.93	0.97	0.92
4	MPXV	Pre	MVA	MVA	Pre	MVA	borderline positive	negative	0	1998	0.58	0.47	0.54
5	MPXV	Pre	MVA	MVA	Pre	Pre	negative	negative		1990	0.8	0.5	0.83
6	MPXV	MPXV	MPXV	MPXV	MPXV	MPXV	borderline positive	positive		1990	1	1	0.89
7	MPXV	MVA	MPXV	MPXV	MPXV	MPXV	positive	positive	1	1980	0.6	0.58	0.66
8	MPXV	MPXV	MPXV	MPXV	MPXV	MPXV	positive	positive	1	1980	1	1	0.83
9	MPXV	MPXV	MPXV	MPXV	MPXV	MPXV	positive	positive	1	1980	1	1	0.85
10	MPXV	MPXV	MPXV	MPXV	MPXV	MPXV	positive	positive	1	1980	1	1	0.83
11	MPXV	MPXV	MPXV	MPXV	MPXV	MPXV	positive	positive	2	1987	0.75	0.75	0.84
12	MPXV	MPXV	MPXV	MPXV	MPXV	MPXV	positive	positive	0	1990	1	1	1
13	MPXV	MPXV	MPXV	MPXV	MPXV	MPXV	positive	positive	0	1990	1	1	1
14	MPXV	MVA	MPXV	MPXV	MVA	MPXV	positive	positive	2	1990	0.53	0.53	0.97
15	MPXV	MVA	MPXV	MPXV	MPXV	MPXV	positive	positive	1	1988	0.5	0.5	0.93
16	MPXV	MVA	MPXV	MPXV	MPXV	MPXV	positive	positive	1	1988	0.52	0.5	0.92
17	MPXV	MVA	MPXV	MPXV	MPXV	MPXV	positive	positive	1	1987	0.5	0.5	0.98
18	MPXV	MPXV	MPXV	MPXV	MPXV	MPXV	positive	positive	0	1974	1	1	0.97
19	MPXV	MPXV	MPXV	MPXV	MPXV	MPXV	positive	positive	0	2000	1	1	0.98
20	MPXV	MPXV	MPXV	MPXV	MPXV	MPXV	positive	positive	0	1974	1	1	0.98
21	MPXV	MVA	MVA	MPXV	MVA	MVA	positive	negative	1	1988	0.82	0.98	0.92
22	MPXV	MVA	MPXV	MPXV	MPXV	MPXV	positive	positive	1	1987	0.52	0.5	0.96
23	MPXV	MVA	MPXV	MPXV	MVA	MPXV	positive	positive	2	1990	0.65	0.55	0.94
24	MPXV	MPXV	MPXV	MPXV	MPXV	MPXV	positive	positive	0	1974	1	1	0.99
25	MPXV	MVA	MPXV	MVA	MVA	MPXV	positive	positive	1	1984	0.58	0.6	0.99
26	MPXV	MVA	MPXV	MPXV	MVA	MPXV	positive	positive	2	1988	0.52	0.52	0.86
27	MPXV	Pre	MVA	Pre	Pre	Pre	negative	negative	2	1987	0.4	0.33	0.75
28	MPXV	Pre	Pre	Pre	Pre	Pre	negative	negative	2	1987	0.5	0.5	0.9
29	MPXV	Pre	Pre	Pre	Pre	Pre	negative	negative	2	1987	0.45	0.42	0.74
30	MPXV	Pre	MVA	MVA	Pre	Pre	negative	negative	2	1988	0.28	0.25	0.69
31	MPXV	MPXV	MPXV	MPXV	MPXV	MPXV	positive	positive	2	1987	1	1	0.83
32	MPXV	MVA	MPXV	MPXV	MPXV	MPXV	positive	positive	2	1988	0.63	0.62	0.73

**Sample**	**Real**	**LDA**	**GBC**	**RF**	**Ensemble mean**	**Ensemble sero**	**Serostatus (Delta)**	**Serostatus ATI-N-CPXV**	**MVA vacc. (n)**	**Year Birth**	**Ensemble conf. serostatus**	**Ensemble conf. mean**	**LDA conf.**
**33**	MPXV	MPXV	MPXV	MPXV	MPXV	MPXV	positive	positive	2	1987	1	1	0.9
**34**	MPXV	MVA	MPXV	MPXV	MVA	MPXV	positive	positive			0.53	0.53	0.95
**35**	MPXV	MVA	MPXV	MPXV	MPXV	MPXV	positive	positive	2	1975	0.5	0.5	0.82
**36**	MPXV	MVA	MPXV	MPXV	MPXV	MPXV	positive	positive	2	1975	0.5	0.5	0.91
**37**	MVA	MVA	Pre	Pre	MVA	MVA	negative	negative	2	1995	0.57	0.53	0.55
**38**	MPXV	MVA	MVA	MPXV	MVA	MVA	positive	positive	2	1995	0.72	0.93	0.97
**39**	MPXV	MVA	MPXV	MPXV	MVA	MPXV	positive	positive	2	1995	0.55	0.72	0.99
**40**	MPXV	MVA	MPXV	MPXV	MPXV	MPXV	positive	positive	2	1978	0.52	0.5	0.9
**41**	MPXV	MVA	Pre	Pre	MVA	MVA	negative	negative	0	1985	0.5	0.5	0.78
**42**	MPXV	MVA	MPXV	MPXV	MVA	MPXV	positive	positive	1	1982	0.53	0.53	0.95
**43**	MPXV	MVA	MPXV	MPXV	MPXV	MPXV	positive	positive	1	1982	0.5	0.5	0.88
**44**	MPXV	MVA	MPXV	MPXV	MPXV	MPXV	positive	positive	1	1982	0.5	0.5	0.93
**45**	MPXV	MVA	MPXV	MPXV	MVA	MPXV	positive	positive	2	1991	0.5	0.5	0.92
**46**	MPXV	Pre	MVA	MVA	MVA	Pre	negative	negative	0	1985	0.48	0.72	0.48
**47**	MPXV	MPXV	MPXV	MPXV	MPXV	MPXV	borderline positive	positive	0	1985	0.9	0.97	0.76
**48**	MPXV	MPXV	MPXV	MPXV	MPXV	MPXV	borderline positive	positive			0.9	0.95	0.76
**49**	MPXV	MPXV	MPXV	MPXV	MPXV	MPXV	positive	positive	0	1985	1	1	0.99
**50**	MPXV	MPXV	MPXV	MPXV	MPXV	MPXV	borderline positive	positive			1	1	0.94
**51**	MPXV	MPXV	MPXV	MPXV	MPXV	MPXV	borderline positive	positive			0.8	0.83	0.76
**52**	MPXV	MVA	MPXV	MPXV	MPXV	MPXV	positive	positive	2	1991	0.5	0.5	0.9
**53**	MPXV	MPXV	MPXV	MPXV	MPXV	MPXV	borderline positive	positive			1	1	0.96
**54**	MPXV	MPXV	MPXV	MPXV	MPXV	MPXV	borderline positive	positive			1	1	0.98
**55**	MPXV	MPXV	MPXV	MPXV	MPXV	MPXV	borderline positive	positive			1	1	0.95
**56**	MPXV	MVA	MPXV	MPXV	MPXV	MPXV	positive	positive			0.5	0.5	0.89
**57**	Pre	MVA	MVA	MVA	MVA	MVA	negative	negative		1990	0.78	1	0.46
**58**	Pre	Pre	MVA	MVA	Pre	Pre	negative	positive		1986	0.72	0.4	0.57
**59**	Pre	Pre	MVA	MVA	Pre	Pre	negative	negative		2002	0.5	0.5	0.63
**60**	Pre	Pre	Pre	Pre	Pre	Pre	negative	negative		1992	1	1	0.89
**61**	Pre	Pre	MPXV	Pre	Pre	Pre	negative	negative		1983	0.65	0.5	0.54
**62**	Pre	MPXV	MVA	MVA	MPXV	MVA	borderline positive	positive		1984	0.45	0.5	0.47
**63**	Pre	Pre	Pre	Pre	Pre	Pre	negative	negative		1997	1	0.9	0.59
**64**	Pre	Pre	MVA	Pre	Pre	Pre	negative	negative		1995	0.83	0.67	0.69
**65**	Pre	MVA	MVA	MVA	MVA	MVA	negative	negative		1987	0.57	0.87	0.41
**66**	Pre	Pre	MVA	MVA	MVA	Pre	negative	negative		1994	0.5	0.5	0.53
**67**	Pre	Pre	Pre	Pre	Pre	Pre	negative	negative		1974	1	1	0.78
**68**	Pre	Pre	Pre	Pre	Pre	Pre	negative	negative		1978	1	0.93	0.7
**69**	Pre	MPXV	MVA	MVA	MVA	MVA	borderline positive	negative		1970	0.75	0.75	0.56
**70**	Pre	MPXV	MVA	MVA	MPXV	MVA	borderline positive	positive		1938	0.57	0.57	0.56
**71**	Pre	Pre	MVA	Pre	Pre	Pre	negative	negative		1999	1	0.7	0.67
**72**	Pre	MPXV	MPXV	MPXV	MPXV	MPXV	positive	negative		1970	0.78	0.82	0.88
**73**	Pre	MVA	MVA	MVA	MVA	MVA	borderline positive	negative		1965	1	1	0.78
**74**	Pre	Pre	Pre	MVA	Pre	Pre	borderline positive	negative		1969	0.88	0.92	0.62

Sample	Real	LDA	GBC	RF	Ensemble mean	Ensemble sero	Serostatus (Delta)	Serostatus ATI-N-CPXV	MVA vacc. (n)	Year Birth	Ensemble conf. serostatus	Ensemble conf. mean	LDA conf.
**75**	Pre	MPXV	MVA	MVA	MVA	MVA	borderline positive	negative		1942	0.87	0.75	0.64
**76**	Pre	MVA	MVA	MVA	MVA	MVA	borderline positive	negative		1960	1	1	0.83
**77**	Pre	MPXV	Pre	MPXV	MPXV	Pre	positive	positive		1945	0.57	0.57	0.87
**78**	Pre	Pre	Pre	MVA	Pre	Pre	borderline positive	negative		1965	0.75	0.97	0.65
**79**	Pre	MVA	MVA	MVA	MVA	MVA	positive	negative		1963	0.87	0.8	0.51
**80**	Pre	MVA	MPXV	MPXV	MVA	MPXV	borderline positive	negative		1949	0.52	0.43	0.5
**81**	Pre	Pre	MVA	MVA	Pre	Pre	negative	negative		1992	0.9	0.5	0.74
**82**	Pre	Pre	Pre	Pre	Pre	Pre	negative	negative		1991	1	0.98	0.67
**83**	Pre	MVA	Pre	MVA	MVA	Pre	borderline positive	negative		1976	0.92	0.67	0.6
**84**	Pre	Pre	Pre	Pre	Pre	Pre	borderline positive	negative		1975	0.77	0.95	0.52
**85**	Pre	Pre	Pre	Pre	Pre	Pre	negative	negative		1982	1	1	0.73
**86**	Pre	MVA	MVA	MVA	MVA	MVA	borderline positive	negative		1934	1	1	0.63
**87**	Pre	Pre	Pre	Pre	Pre	Pre	negative	negative		1975	1	0.92	0.76
**88**	Pre	Pre	Pre	Pre	Pre	Pre	negative	negative		1984	1	1	0.79
**89**	Pre	MVA	MVA	MVA	MVA	MVA	positive	negative		1963	0.92	0.7	0.48
**90**	Pre	Pre	MVA	MVA	Pre	Pre	negative	negative		1947	0.52	0.52	0.68
**91**	Pre	Pre	Pre	MVA	Pre	Pre	positive	negative		1948	0.52	0.58	0.49
**92**	Pre	MVA	MVA	MVA	MVA	MVA	borderline positive	negative		1941	1	1	0.69
**93**	Pre	Pre	Pre	MVA	Pre	Pre	borderline positive	negative		1955	0.65	0.75	0.47
**94**	Pre	MPXV	MVA	MVA	MPXV	MVA	borderline positive	positive		1947	0.52	0.65	0.77
**95**	Pre	MVA	Pre	Pre	MVA	MVA	negative	negative		1984	0.83	0.52	0.53
**96**	Pre	Pre	MVA	MVA	MVA	Pre	negative	negative		1982	0.47	0.48	0.48
**97**	Pre	Pre	Pre	Pre	Pre	Pre	negative	negative		1983	1	1	0.79
**98**	Pre	MVA	MVA	MVA	MVA	MVA	negative	negative		1980	0.78	0.85	0.93
**99**	Pre	MVA	MVA	MVA	MVA	MVA	borderline positive	negative		1996	0.77	0.93	0.52
**100**	Pre	Pre	Pre	Pre	Pre	Pre	negative	negative		1974	0.98	0.92	0.63
**101**	Pre	Pre	Pre	Pre	Pre	Pre	negative	negative		1969	0.98	0.85	0.61
**102**	Pre	Pre	Pre	Pre	Pre	Pre	negative	negative		1980	1	1	0.81
**103**	Pre	Pre	Pre	Pre	Pre	Pre	negative	negative		1993	1	1	0.89
**104**	Pre	Pre	Pre	Pre	Pre	Pre	borderline positive	negative		1967	0.55	0.78	0.63
**105**	Pre	MVA	MVA	MVA	MVA	MVA	positive	negative		1963	0.9	0.83	0.86
**106**	Pre	MVA	Pre	Pre	Pre	Pre	positive	negative		1966	0.58	0.53	0.51
**107**	MVA	MVA	MVA	MVA	MVA	MVA	borderline positive	negative	3	1984	0.92	0.92	1
**108**	MVA	MVA	MVA	MVA	MVA	MVA	borderline positive	negative	3	1982	1	1	0.89
**109**	MVA	MVA	MVA	MVA	MVA	MVA	positive	negative	3	1982	1	1	0.99
**110**	MVA	MVA	MVA	MVA	MVA	MVA	borderline positive	negative	3	1985	1	1	0.95
**111**	MVA	MVA	MVA	MVA	MVA	MVA	borderline positive	negative	3	1985	1	1	0.91
**112**	MVA	MVA	MVA	MVA	MVA	MVA	borderline negative	negative	3	1985	1	1	0.93
**113**	MVA	MVA	MVA	MVA	MVA	MVA	positive	negative	3	1983	1	1	1
**114**	MVA	MVA	MVA	MVA	MVA	MVA	borderline positive	negative	3	1983	1	1	0.99
**115**	MVA	MVA	MVA	MVA	MVA	MVA	positive	negative	2	1983	1	1	0.95
**116**	MVA	MVA	MVA	MVA	MVA	MVA	positive	negative	2	1964	1	1	0.99

Sample	Real	LDA	GBC	RF	Ensemble mean	Ensemble sero	Serostatus (Delta)	Serostatus ATI-N-CPXV	MVA vacc. (n)	Year Birth	Ensemble conf. serostatus	Ensemble conf. mean	LDA conf.
**117**	MVA	MVA	MVA	MVA	MVA	MVA	borderline positive	negative	2	1964	1	1	0.95
**118**	MVA	MVA	MVA	MVA	MVA	MVA	negative	negative	2	1981	1	1	0.77
**119**	MVA	MVA	MVA	MVA	MVA	MVA	positive	negative	2	1981	1	1	1
**120**	MVA	MVA	MVA	MVA	MVA	MVA	positive	negative	3	1973	1	1	0.94
**121**	MVA	MVA	MVA	MVA	MVA	MVA	positive	negative	3	1973	1	1	0.93
**122**	MVA	MVA	MVA	MVA	MVA	MVA	positive	negative	3	1971	1	1	0.95
**123**	MVA	MVA	MVA	MVA	MVA	MVA	positive	negative	4	1971	1	1	0.96
**124**	MVA	MVA	MPXV	MVA	MVA	MPXV	borderline positive	positive	2	1965	0.65	0.6	0.48
**125**	MVA	MVA	MVA	MVA	MVA	MVA	borderline positive	positive	2	1965	0.82	0.77	0.78
**126**	MVA	MVA	MVA	MVA	MVA	MVA	positive	negative	2	1966	1	1	1
**127**	MVA	MVA	MVA	MVA	MVA	MVA	positive	negative	3	1984	1	1	1
**128**	MVA	MVA	MVA	MVA	MVA	MVA	positive	negative	3	1984	1	1	0.99
**129**	MVA	MVA	MVA	MVA	MVA	MVA	positive	negative	4	1984	1	1	0.99
**130**	MVA	Pre	MVA	MVA	MVA	Pre	negative	negative	1	1987	0.75	0.75	0.57
**131**	MVA	MVA	MVA	MVA	MVA	MVA	negative	negative	2	1987	0.78	0.8	0.49
**132**	MVA	MVA	MVA	MVA	MVA	MVA	negative	negative	1	1994	1	1	0.74
**133**	MVA	MVA	MVA	MVA	MVA	MVA	negative	negative	2	1994	1	1	0.86
**134**	MVA	MVA	MVA	MVA	MVA	MVA	borderline positive	negative	2	1987	0.98	1	0.85
**135**	MVA	Pre	MVA	MVA	Pre	Pre	negative	positive	2	1987	0.52	0.5	0.56
**136**	MVA	MVA	MVA	MVA	MVA	MVA	borderline positive	negative	2	1999	1	1	0.88
**137**	MVA	MVA	MVA	MVA	MVA	MVA	negative	negative	2	1999	1	1	0.5
**138**	Pre	MVA	Pre	MVA	MVA	Pre	positive	negative		1959	0.73	0.67	0.77
**139**	MVA	MVA	MVA	MVA	MVA	MVA	negative	negative		1978	1	1	0.7
**140**	Pre	Pre	Pre	Pre	Pre	Pre	negative	negative		1997	1	1	0.78
**141**	MVA	Pre	MVA	MVA	Pre	Pre	negative	negative		1997	0.52	0.5	0.67
**142**	Pre	Pre	MVA	MVA	Pre	Pre	negative	negative		1996	0.52	0.38	0.53
**143**	MVA	MVA	MVA	MVA	MVA	MVA	borderline positive	negative		1996	0.85	0.98	0.73

LDA, Linear Discriminant Analysis; GBC, Gradient Boosting Classifier; RF, Random Forest; IF, immunofluorescence assay; pre, pre-immune; VACV, vaccinia virus; CPXV, cowpox virus; MVA, modified vaccinia ankara.

Supplementary Table 11. Class-wise sensitivities and specificities (mean and 95% CI from bootstrap analysis with 2000 iterations) for GBC ensemble predictions, for all data or only confident predictions (ensemble confidence score > 0.5) of the combined serological cohort or the independent validation cohort. Source data are provided as a Source Data file.

Class	Filtered	Cohort	Sensitivity	Specificity
**MPXV**	All	Combined	0.86 (0.83–0.90)	0.90 (0.88–0.92)
**MPXV**	All	Independent Validation	0.92 (0.83–0.98)	0.88 (0.82–0.95)
**MPXV**	Confident predictions	Combined	0.90 (0.86–0.93)	0.92 (0.90–0.93)
**MPXV**	Confident predictions	Independent Validation	0.94 (0.86–1.00)	0.93 (0.88–0.99)
**MVA**	All	Combined	0.85 (0.82–0.88)	0.92 (0.90–0.94)
**MVA**	All	Independent Validation	0.50 (0.38–0.63)	0.97 (0.93–1.00)
**MVA**	Confident predictions	Combined	0.85 (0.82–0.88)	0.93 (0.91–0.95)
**MVA**	Confident predictions	Independent Validation	0.58 (0.44–0.71)	0.97 (0.93–1.00)
**Pre**	All	Combined	0.78 (0.74–0.82)	0.92 (0.90–0.94)
**Pre**	All	Independent Validation	0.86 (0.71–0.97)	0.75 (0.67–0.83)
**Pre**	Confident predictions	Combined	0.82 (0.78–0.85)	0.93 (0.91–0.94)
**Pre**	Confident predictions	Independent Validation	0.92 (0.79–1.00)	0.80 (0.72–0.87)

MPXV, mpox virus; MVA, modified vaccinia ankara; Pre, pre-immune.

Supplementary Table 12. Performance characteristics (sensitivity, specificity, accuracy) and number of sera (true positive tp, true negative tn, false negative fn, false positive fp) for ROC analysis to determine threshold values (log_{10} quantified IgG) to discern seropositive from seronegative samples (serostatus) or MPXV infected from non-infected samples based on single antigens.

Antigen	Class	Threshold	Sensitivity	Specificity	Accuracy	tn	tp	fn	fp	n
**A35-MPXV**	Infected	2.83389625	0.67	0.72	0.70	368	244	119	144	875
**ATI-N-CPXV**	Infected	2.094029684	0.71	0.85	0.79	437	257	106	75	875
**B6-MPXV**	Infected	2.548108799	0.60	0.72	0.67	369	218	145	143	875
**E8-MPXV**	Infected	3.1892547	0.50	0.70	0.61	357	181	181	155	875
**A29/A27**	Infected	0.894713751	0.67	0.60	0.63	307	243	120	205	875
**A35/A33**	Infected	1.020428581	0.74	0.82	0.79	420	267	96	92	875
**B6/B5**	Infected	0.97050582	0.61	0.61	0.61	312	223	140	199	875
**E8/D8**	Infected	1.029724974	0.64	0.78	0.72	400	232	131	112	875
**M1/L1**	Infected	1.025193225	0.63	0.56	0.59	285	228	135	227	875
**A27-VACV**	Serostatus	2.615532835	0.65	0.57	0.62	152	331	178	114	775
**A29-MPXV**	Serostatus	2.378226547	0.57	0.68	0.61	182	288	221	84	775
**A33-VACV**	Serostatus	2.14692063	0.74	0.76	0.75	201	379	130	65	775
**A35-MPXV**	Serostatus	2.078120047	0.74	0.82	0.77	219	378	131	47	775
**A5-CPXV**	Serostatus	3.504355774	0.61	0.56	0.59	149	312	197	117	775
**ATI-C-CPXV**	Serostatus	3.547389762	0.59	0.62	0.61	164	302	207	102	775
**ATI-N-CPXV**	Serostatus	1.906636067	0.50	0.67	0.56	178	253	256	88	775
**B5-VACV**	Serostatus	1.984324057	0.71	0.79	0.74	209	362	147	57	775
**B6-MPXV**	Serostatus	1.540443056	0.83	0.81	0.82	215	422	87	51	775
**D8-VACV**	Serostatus	2.397074248	0.76	0.80	0.77	213	387	122	53	775
**Delta-VACV**	Serostatus	1.486705187	0.76	0.82	0.78	219	388	121	47	775
**E8-VACV**	Serostatus	1.991973072	0.84	0.86	0.85	229	427	82	37	775
**H3-VACV**	Serostatus	2.740489908	0.66	0.55	0.62	145	335	174	121	775
**L1-VACV**	Serostatus	3.214781184	0.62	0.63	0.62	168	315	194	98	775
**M1-VACV**	Serostatus	3.354378744	0.60	0.58	0.60	154	307	202	112	775

ROC, Receiver Operator Characteristic; MPXV, mpox virus; CPXV, cowpox virus; VACV, vaccinia virus.

Supplementary Table 13. Comparison of performance parameters for prediction of MPXV infection by single antigens using a classical ROC-based approach or GBC

Cohort	Classes†	Antigens Classical‡	Classical F1 (95% CI)	ML F1 (95% CI)	ML vs. Classical: Difference (ML–Classical)#
Combined	Binary Infected	ATI-N-CPXV	0.68 (0.64–0.71)	0.80 (0.77–0.83)	**+0.12 (significant)**
		A35/A33 Ratio	0.68 (0.64–0.72)		**+0.12 (significant)**
Combined	Binary Serostatus	E8-MPXV	0.85 (0.83–0.87)	0.90 (0.89–0.92)	**+0.05 (significant)**
		B6-MPXV	0.85 (0.83–0.87)		**+0.05 (significant)**
Combined	Multi	ATI-N-CPXV + E8-MPXV	0.72 (0.70–0.75)	0.82 (0.80–0.85)	**+0.10 (significant)**
		A35/A33 Ratio + B6-MPXV	0.70 (0.68–0.73)		**+0.12 (significant)**
Validation	Binary Infected	ATI-N-CPXV	0.83 (0.75–0.90)	0.85 (0.78–0.92)	+0.02 (not significant)
		A35/A33 Ratio	0.65 (0.53–0.75)		**+0.20 (significant)**
Validation	Binary Serostatus	E8-MPXV	0.82 (0.76–0.87)	0.84 (0.79–0.89)	+0.02 (not significant)
		B6-MPXV	0.82 (0.76–0.87)		+0.02 (not significant)
Validation	Multi	ATI-N-CPXV + E8-MPXV	0.65 (0.57–0.72)	0.70 (0.63–0.77)	+0.05 (not significant)
		A35/A33 Ratio + B6-MPXV	0.54 (0.46–0.62)		**+0.16 (significant)**

† Binary classification refers to distinguishing either "Mpox" versus "Non-infected" (Binary Infected), or "Positive" versus "Negative" (Binary Serostatus). Multi-class classification includes the categories "Pre," "MVA," and "Mpox."

‡ For binary classification, performance is reported for single-antigen approaches: ATI-N-CPXV or ratios of binding to A35-MPXV/A33-VACV (Binary Infected) or E8-MPXV or B6-MPXV (Binary Serostatus). For multi-class classification, the specified combinations have been used.

# Statistical significance was determined by non-overlapping 95% confidence intervals, corresponding to significance at the 5% level. MPXV, mpox virus; ROC, Receiver Operator Characteristic; GBC, Gradient Boosting Classifier; CPXV, cowpox virus.

Supplementary Table 14. Macro-F1 scores of ensemble GBC classifiers across classification tasks, evaluated on the combined cohort (acute + epidemiological) and the independent validation cohort. Models were trained on the combined dataset using either the full antigen panel (baseline) or with individual/multiple antigens excluded (e.g., ATI-N-CPXV, E8-MPXV, D8-VACV).

Antigens excluded	Cohort	Classes†	F1 (95% CI) ‡
**Baseline**	Combined	Binary Infected	**0.80 (0.77–0.83)**
**ATI-N**	Combined	Binary Infected	0.82 (0.79–0.85)
**D8**	Combined	Binary Infected	0.81 (0.78–0.84)
**E8**	Combined	Binary Infected	0.80 (0.77–0.83)
**ATI-N E8**	Combined	Binary Infected	0.81 (0.78–0.84)
**ATI-N E8 D8**	Combined	Binary Infected	0.80 (0.76–0.83)
**Baseline**	Combined	Binary Serostatus	**0.90 (0.89–0.92)**
**ATI-N**	Combined	Binary Serostatus	0.90 (0.88–0.91)
**D8**	Combined	Binary Serostatus	0.90 (0.88–0.91)
**E8**	Combined	Binary Serostatus	0.90 (0.88–0.91)
**ATI-N E8**	Combined	Binary Serostatus	0.89 (0.88–0.91)
**ATI-N E8 D8**	Combined	Binary Serostatus	0.87 (0.85–0.89)
**Baseline**	Combined	Multi	**0.82 (0.80–0.85)**
**ATI-N**	Combined	Multi	0.82 (0.80–0.84)
**D8**	Combined	Multi	0.82 (0.80–0.85)
**E8**	Combined	Multi	0.81 (0.79–0.83)
**ATI-N E8**	Combined	Multi	0.81 (0.79–0.83)
**ATI-N E8 D8**	Combined	Multi	0.78 (0.75–0.80)
**Baseline**	Validation	Binary Infected	**0.85 (0.77–0.92)**
**ATI-N**	Validation	Binary Infected	0.85 (0.78–0.92)
**D8**	Validation	Binary Infected	0.82 (0.74–0.90)
**E8**	Validation	Binary Infected	0.83 (0.74–0.90)
**ATI-N E8**	Validation	Binary Infected	0.85 (0.77–0.91)
**ATI-N E8 D8**	Validation	Binary Infected	0.85 (0.77–0.91)
**Baseline**	Validation	Binary Serostatus	**0.84 (0.79–0.89)**
**ATI-N**	Validation	Binary Serostatus	0.85 (0.80–0.90)
**D8**	Validation	Binary Serostatus	0.85 (0.80–0.90)
**E8**	Validation	Binary Serostatus	0.85 (0.80–0.90)
**ATI-N E8**	Validation	Binary Serostatus	0.85 (0.80–0.90)
**ATI-N E8 D8**	Validation	Binary Serostatus	0.82 (0.76–0.88)
**Baseline**	Validation	Multi	**0.70 (0.63–0.78)**
**ATI-N**	Validation	Multi	0.74 (0.68–0.81)
**D8**	Validation	Multi	0.71 (0.64–0.78)
**E8**	Validation	Multi	0.72 (0.65–0.79)
**ATI-N E8**	Validation	Multi	0.76 (0.69–0.83)
**ATI-N E8 D8**	Validation	Multi	0.69 (0.62–0.76)

† Binary classification refers to distinguishing either “Mpox” versus “Non-infected” (Binary Infected), or “Positive” versus “Negative” (Binary Serostatus). Multi-class classification includes the categories “Pre,” “MVA,” and “Mpox.”

‡ No model was significantly different based on non-overlapping 95% confidence intervals compared to the baseline F1 scores.

GBC, Gradient Boosting Classifier; CPXV, cowpox virus; MPXV, mpox virus; VACV, vaccinia virus.

Supplementary Table 15. Antibodies and detection reagents used to establish a 19-plex serological suspension-assay for detection of the humoral immune response against orthopoxviruses.

Type	Antibody/Reagent	Label	Host	Catalog No.	Source
**Secondary antibody**	Anti-human IgG (Fc-γ specific)	PE-labelled	Goat	109-115-098	Dianova, Hamburg, Germany
**Secondary antibody**	Anti-human IgG (Fc-γ specific)	Unlabelled	Goat	109-005-008	Dianova
**Secondary antibody**	Anti-human IgM (Fc-μ specific)	Unlabelled	Goat	109-005-129	Dianova
**Secondary antibody**	Anti-human IgM (Fc-μ specific)	PE-labelled	Donkey	709-116-073	Dianova
**Secondary antibody**	Anti-mouse IgG (Fc-γ specific)	PE-labelled	Goat	-	Dianova
**Primary antibody**	Anti-A27-VACV (A1/40)	-	Mouse (monoclonal)	-	In house[1]
**Primary antibody**	Anti-D8-VACV	Biotinylated	Goat (polyclonal)	-	In house[1]
**Primary antibody**	Anti-H3-VACV	Biotinylated	Goat (polyclonal)	-	In house[1]
**Primary antibody**	Anti-L1-VACV	Biotinylated	Rabbit (polyclonal)	-	In house[1]
**Primary antibody**	Anti-A33-VACV	Biotinylated	Rabbit (polyclonal)	-	Open Biosystems (Thermo Fisher), Germany
**Primary antibody**	Anti-6×His	-	Mouse (monoclonal)	TD264974	Invitrogen (Thermo Fisher), Germany
**Detection reagent**	Streptavidin-R-Phycoerythrin (SA-PE)	PE	-	PJRS27	Agilent, Santa Clara, CA, USA
**Polyclonal reference antibody**	Anti-Vaccinia virus (VIG)	-	Human	NR-2632	NIH BEI Resources (NIAID, NIH)
**Monoclonal reference antibody**	Anti-B5-VACV (WR, ectodomain)	-	-	NR-429	NIH BEI Resources (NIAID, NIH)

Supplementary Table 16. Antigens used to establish a 19-plex serological suspension-assay for detection of the humoral immune response against orthopoxviruses.

Viral strain	Target protein	Supplier/Source	Order number	Sequence (aa)	Host	Reference strain	Calculated MW (kDa)†	Coupling amount (µg)	Bead Region
**VACV**	A27-VACV	BEI Resources	NR-22133	1-110	Sf9 insect cells	Western Reserve, P11258.3	13.6	20	89
**MPXV**	A29-MPXV	ProteoGenix	PX-P6057	2-110	Mammalian Cells	Zaire-96-I-16, Q77HM6	15.3	10	55
**VACV**	L1-VACV	BEI Resources	NR-21986	1-185	High Five™ insect cells	Western Reserve, P07612	20.8	10	15
**MPXV**	M1-MPXV	ProteoGenix	PX-P6056	2-183	Mammalian Cells	Zaire-96-I-16, Q8V502	22.4	10	36
**VACV**	D8-VACV	Custom Expression	–	2-260	*E.* coli	NYBOH, WR#: P04195	29.9	10	7
**MPXV**	E8-MPXV	ProteoGenix	PX-P6055	2-274	Mammalian Cells	Zaire-96-I-16, AAL40563.1	34.9	10	52
**VACV**	H3-VACV	Custom Expression	–	21-270	*E.* coli	NYBOH, WR#: P07240	29.2	10	59
**VACV**	A33-VACV	BEI Resources	NR-2623	58-185	Cabbage looper insect larvae	Western Reserve, P68617.1	15.1	10	33
**MPXV**	A35-MPXV	ProteoGenix	PX-P6034	57-181	Mammalian Cells	Zaire-96-I-16, AAL40603.1	16.6	10	81
**VACV**	B5-VACV	BEI Resources	NR-22132	20-275	Sf9 insect cells	Western Reserve, Q01227.1	29.4	10	26
**MPXV**	B6-MPXV	ProteoGenix	PX-P6031	20-274	Mammalian Cells	Zaire-96-I-16, AAL40625.1	31.7	10	42
**CPXV**	A5-CPXV	Custom Expression	–	1-292	*E.* coli	GuWi2 BR#: Q8QMU5	32.3	10	54
**CPXV**	ATI-N-CPXV	Custom Expression	–	1-725	*E.* coli	GuWi2 BR#: Q8QMS9	84.6	10	38
**CPXV**	ATI-C-CPXV	Custom Expression	–	725-1284	*E.* coli	GuWi2 BR#: Q8QMS9	65.8	10	82
**VACV**	Cell Lysate	In-house	–	–	Mammalian Cells	NYCBOH, ATCC: VR-1536	–	40	48
–	Cell Lysate	In-house	–	–	Mammalian Cells	HEp-2, ATCC: CCL-23™	–	40	67
–	Human serum albumin	Merck Sigma-Aldrich® (Purified)	A9511-500mg	–	–	–	66	40	8
–	Goat Anti-human IgG (Fc)	Dianova	109-005-008	–	–	–	150	40	20
–	Goat Anti-human IgM (µ)	Dianova	109-005-129	–	–	–	150	40	76

Reference ID for strain Brighton Red (CPXV) or Western Reserve (VACV)
† Based on supplier information (ProteoGenix) or calculated using Expasy ProtParam
MW, molecular weight; kDa, kilodaltons; aa, amino acids; VACV, vaccinia virus; MPXV, mpox virus; CPXV, cowpox virus.

Supplementary Table 17. Expression vector, primers, restriction sites and E. coli strain used for expression of ATI-N (C- and N-terminal domains) and A5.

Protein	Expression vector	Primer	Sequence	Restriction sites	*E. coli* strain
ATI-N-CPXV	pQE-100 S	F (5′→3′)	CGTCGGCATATGCAACGTCTTAGAGAT	NdeI / NsiI	BL21 (DE3) Codon Plus RIPL (Agilent)
		R (5′→3′)	CCTGTTATGCATAACCGTCTTTTCTGGC		
ATI-C-CPXV	pQE-100 S	F (5′→3′)	CCTGTTGGATCCATGGAGGTCACGAACCT	BamHI / HindIII	Rosetta (DE3) pLysS (Novagen)
		R (5′→3′)	AACAGGAAGCTTTTACATCTCTCTTTCTGTATCG		
A5-CPXV	pQE-100 S	F (5′→3′)	CGTCGGCATATGGACTTCTTTAACA	NdeI / NsiI	BL21 (DE3) (Agilent)
		R (5′→3′)	CCTGTTATGCATCTTTTGGAATCGTTCA		

Supplementary methods

Determination of sequence identity and purity of recombinant proteins by LC-MS/MS analysis

Experimental procedure

Proteins were analysed by high-resolution tandem mass spectrometry (MS/MS) according to an earlier published protocol with slight modifications [3]. Briefly, samples were diluted to 100 pmol in 50 mM ammonium bicarbonate buffer with 9% (v/v) acetonitrile and reduced by adding 1.5 µL of 400 mM dithiothreitol (DTT, Sigma-Aldrich, Munich, Germany) for 10 min at 95 °C. Alkylation was achieved by adding 3 µL of 500 mM iodoacetamide (IAA, Sigma-Aldrich, Munich, Germany) and incubated for 30 min at 37 °C. Tryptic digest was performed by adding 10 µL of 0.02 µg/µL proteomics grade trypsin solution (Sigma-Aldrich, Munich, Germany) and incubated overnight at 37 °C. Reaction was stopped by adding 4 µL of 10% trifluoroacetic acid (TFA, Merck, Darmstadt, Germany). Peptides were desalted and purified using C18 ZipTip (Merck, Darmstadt, Germany) according to the manufacturer's protocol. ZipTip eluate was dried in a speedvac concentrator and resuspended in 15 µL of 0.1% formic acid (Thermo Scientific, Bremen, Germany). Concentration of resulting peptides was determined by absorbance measurement at 280 nm in a NanoPhotometer (Thermo Fisher Scientific, Bremen, Germany). Peptides were analyzed on a nanoLC (EASY-nanoLC 1200, Thermo Fisher Scientific, Bremen, Germany) coupled online to an Orbitrap mass spectrometer (Q Exactive™ HF, Thermo Fisher Scientific, Bremen). Peptide solution (5 µL) was loaded and separated on a ReproSil C18 PepSep column (15 cm length, 75 µm i.d., 100 Å, 1.5 µm; PepSep, Marslev, Denmark) using a 30 min gradient of 4% to 38% acetonitrile in 0.1% of formic acid at a 300 nL/min flow rate. The temperature of the LC column was set to 50 °C. The mass spectrometer was operated in a data-dependent acquisition mode (DDA) and the following settings were applied: full scan spectra (MS1) were recorded with a scan resolution of 60,000 in a scan range of 300 to 1650 m/z. The MS^1 automatic gain control (AGC) target value was set to 3×10^6 with a maximum injection time of 20 ms. Fragment spectra (MS^2) were obtained by higher-energy c-trap dissociation (HCD) with a normalized collision energy (NCE) of 27% for up to the 10 most intense 2+ to 5+ charged ions. MS2 scan resolution was 30,000 at 200 m/z. MS^2 AGC target value was set to 1×10^5 with a maximum injection time of 50 ms and an isolation window of 2.0 m/z. The minimum AGC target value was set to 1×10^4 and a dynamic exclusion of 30 s within a 10 ppm window. Peptides were ionized using electrospray with a stainless-steel emitter, i.d. 30 µm, (PepSep, Marslev, Denmark) at a spray voltage of 2.1 kV and a heated capillary temperature of 275 °C. Mass data were processed by Proteome Discover software (Thermo Fisher Scientific, Bremen, Germany) as well as MASCOT server 2.4 software (Matrix Science Ltd., London, UK). Database search was done with carbamidomethyl (C) as fixed and oxidation (M) as variable modification. Protein mass was unrestricted, the peptide mass tolerance was set to ± 10 ppm while the fragment mass tolerance was set to ± 0.02 Da. A maximum number of two missed tryptic cleavages were allowed. Supplier provided protein sequences or sequences from the UniProt database[4] were used as database entries for peptide identification. Identification and coverage of the reference sequence was calculated. Although the method is not quantitative, major contaminants as characterized by a large number of identified peptides against the whole UniProt database are also mentioned where applicable. Due to the high sensitivity of the method, trace amounts of contaminants can also be identified. Most contaminants that could be found were derived from the respective host expression vector.

Identified peptide sequences

The following list contains the protein sequence of the analyzed proteins with identified peptide sequences marked in **bold and red**. Besides the reference sequence that was employed for sequence alignment and the protein sequence coverage in percent of the reference sequence, remarks on identified contaminants is also added for each protein analysed.

Protein: A5-CPXV
Reference sequence: P29191, 39kDa core protein OS=Vaccinia virus (strain Western Reserve)
Protein sequence coverage: 35%

1 **MDFFNKFSQG LAESSTPKSS IYYSEEKDPD TKKDEAIEIG LKSQESYYQR**
51 **QLREQLAR**DN MTVASRQPIQ PLQPTIHITP QPVPTATPAP ILLPSSTVPT
101 PKPRQQTNTS SDMSNLFDWL SEDTDAPASS LLPALTPSNA VQDIISKFNK
151 DQKTTTPPST QPSQTLPTTT CTQQSDGNIS CTTPTVTPPQ PPIVATVCTP
201 TPTGGTVCTT AQQNPNPGAA SQQNLDDMAL K**DLMSNVERD MHQLQAETND**
251 **LVTNVYDAR**E YTR**RAIDQIL QLVKGFER**FQ K

Remarks/ Contamination: Large number of *E. coli* proteins.

Protein: A27-VACV
Reference sequence: P11258, Protein A27 OS=Vaccinia virus (strain Western Reserve)
Protein sequence coverage: 68%

1 MDGTLFPGDD DLAIPATEFF STKAAKKPEA K**REAIVKADE DDNEETLKQR**
51 **LTNLEKKITN VTTKFEQIEK CCKRNDEVLF RLENHAETLR AAMISLAKKI**
101 **DVQTGR**RPYE

Reference sequence: A27-VACV + His-Tag (provided by supplier)
Protein sequence coverage: 63%

1 DPMDGTLFPG DDDLAIPATE FFSTKAAKKP EAK**REAIVKA DEDDNEETLK**
51 **QRLTNLEKKI TNVTTKFEQI EKCCKRNDEV LFRLENHAET LRAAMISLAK**
101 **KIDVQTGR**RP YEHHHHHH

Remarks/ Contamination: High purity, identification of some baculovirus (*Autographa californica nuclear polyhedrosis virus*) proteins.

Protein: A29-MPXV
Reference sequence: P11258, Protein A27 OS=Vaccinia virus (strain Western Reserve)
Protein sequence coverage: 72%

1 M**DGTLFPGDD DLAIPATEFF STKAAK**KPEA KR**EAIVK**ADE DDNEETLK**QR**
51 **LTNLEKK**ITN VTTK**FEQIEK CCK**R**NDEVLF RLENHAETLR AAMISLAKKI**
101 **DVQTGR**RPYE

Reference sequence: A29-MPXV Asp2-Glu110 (provided by supplier)
Protein sequence coverage: 95%

1 M**DGTLFPGDD DLAIPATEFF STKAAKNPET KREAIVKAYG DDNEETLKQR**
51 **LTNLEKKITN ITTKFEQIEK CCKHNDEVLF RLENHAETLR AAMISLAKKI**
101 **DVQTGR**RPYE

Remarks/ Contamination: Large number of human proteins (expression host HEK293).

Protein: A33-VACV
Reference sequence: P68616, Protein A33 OS=Vaccinia virus (strain Copenhagen)
Protein sequence coverage: 53%

1 MMTPENDEEQ TSVFSATVYG DKIQGKNKRK RVIGLCIRIS MVISLLSMIT
51 MSAFLIVR**LN QCMSANEAAI TDAAVAVAAA SSTHRKVASS TTQYDHKESC**
101 **NGLYYQGSCY ILHSDYQLFS DAKANCTAES STLPNK**SDVL ITWLIDYVED

151 TWGSDGNPIT K**TTSDYQDSD VSQEVRKYFC VK**TMN

Reference sequence: A33-VACV Arg58- Asn185 (provided by supplier)

Protein sequence coverage: 81%

1 **DPRLNQCMSA NEAAITDAAV AVAAASSTHR KVASSTTQYD HKESCNGLYY**

51 **QGSCYILHSD YQLFSDAKAN CTAESSTLPN K**SDVLITWLI DYVEDTWGSD

101 GNPITK**TTSD YQDSDVSQEV RKYFCVKTMN HHHHHH**

Remarks/ Contamination: Highly pure protein, only minor contaminations

Protein: A35-MPXV

Reference sequence: P68616, Protein A33 OS=Vaccinia virus (strain Copenhagen)

Protein sequence coverage: 29%

1 MMTPENDEEQ TSVFSATVYG DKIQGKNKRK RVIGLCIRIS MVISLLSMIT

51 MSAFLIVR**LN QCMSANEAAI TDAAVAVAAA SSTHRKVASS TTQYDHK**ESC

101 NGLYYQGSCY ILHSDYQLFS DAKANCTAES STLPNKSDVL ITWLIDYVED

151 TWGSDGNPIT K**TTSDYQDSD VSQEVRK**YFC VKTMN

Reference sequence: A35-MPXV Val57-Thr181 (provided by supplier)

Protein sequence coverage: 67% (100% of expressed fragment)

1 MMTPENDEEQ TSVFSATVYG DKIQGKNKRK RVIGLCIRIS MVISLLSMIT

51 MSAFLIVR**QN QCMSANEAAI TDSAVAVAAA SSTHRKVASS TTQYDHKESC**

101 **NGLYYQGSCY ILHSDYKSFE DAKANCAAES STLPNKSDVL TTWLIDYVED**

151 **TWGSDGNPIT KTTSDYQDSD VSQEVRKYFC T**

Remarks/ Contamination: Large number of human proteins (expression host HEK293).

Protein: ATI-C-CPXV

Reference sequence: P16602, A-type inclusion protein A25 homolog OS=Cowpox virus

Protein sequence coverage: 28%

1 MEVTNLIEKC TKHSKDFATE VEKLWNDELS SESGLSRKTR NVIRNILRDI

51 TKSLTTDKKS KCFRILERST INGEQIKDVY KTIFNNGVDV ESRINTTGKY

101 VLFTVMTYAA AELRLIKSDE IFALLSRFFN MICDIHRKYG CGNMFVGIPA

151 ALINLLEIDQ INKLFSVFST RYDAKTFIYT EYFLFLNINH YLLVSGSELF

201 INVAYGPASF SSPISVPDYI MEALTFKACD HIMKSGDLKY TYAFTKKVKD

251 LFNTKSDSVY QYVRLHEMSY DGVSEDTDDD DEVFAILNLS IDSSVDRYRN

301 RVLLLTPEVA SLRKEYSDVE PDYKYLMDEE VPAYDKHLPK PITNTGIEEP

351 HATGGDKEEQ EQQPVKVVQS KPDDGITPYN PFEDPDYVPT ITKTVLGIAD

401 YQLVINKLIE WLDKCEEECG NGGEYKTELE EAKRKLTELN LELSDKLSKI

451 RTLERDSVYK TERIDRLTKE IKELRDIQNG TDDGSDSSEI DKKTIRELRE

501 SLDREREMRT ELERELDTIR DGKVEGSCQR ELELSRMWLK QRDDDLRAEI

551 DKRRNVEWEL SRLRRDIKEC DKYKEDLDKA KTTISNYVSR ISTLESEIAK

601 YQQDRDTLSV VRRELEEERR RVRDLESRLD ECTRNQEDTQ EVDALRSRIR

651 ELENKLADCM ESGGGNLTEI SRLQSK**ISDL ER**QLRECRGN ATEISRLQYR

701 **ITDLER**QLND CRRNNENNAD TEREMQRLRD RITDLERQLS DCRRNNESNA

751 DMEREMQR**LR DRIMDLDRQL NECKRDGNGT SSEEVNRLKT RIRDLERSLE**

801 **ICSKDESELY SAYKSELGRA REQISNLQES LRRERESDKT DSYYR**RELTR

851 **ERNKIVELEK** **ELNKCFDTNH** **AK**YIDEINSK K**TRISDLERQ** **LAACKSNGGS**
901 **NGDMDQYKRE** **IESLKRELAE** **CRRGNNGSHS** **DCKYYDEEAR** **EEVKRLRQEL**
951 **TQLHEDLKRA** **RESDKNDSYY** **KRELERQRAK** **VIEVEKELER** YFDDSRLAEC
1001 K**RHGDEMLRK** **IADLEKKLR**D GGNGNGGNGC TSSCEFERKR IAVLEVEVR**K**
1051 **SMETIKSLEK** **FMEFDRLQKD** **CADKLDREK**E RR**MKAERDLE** **REIARKNCGG**
1101 **NPCERELESE** **RSNVKRLEYQ** **LDAEKEKVKF** **YKRELERDRY** **LSSRYLTSSS**
1151 **DPDEKPLPNY** **TFPR**IEVEPL TTEDTEPKPV EVVPPSSDVT EPISSGVTPS
1201 VDAEPEHPQL SEYQTSVSQV AVTPPPKPET PQISEYQDYS ELYSASNNTE
1251 SKNVFSELAY LDDLDKLDDI DEYLLNNIMP EKTV

Reference sequence: Q80HU9, 27 kDa A-type inclusion protein OS=Vaccinia virus (strain Western Reserve)
Protein sequence coverage: 62%

1 MKPMPKQREM RRLRDR**ISDI** **ER**QLSDCRRN NESNADMERE MQR**LRDRIMD**
51 **LDRQLNECKR** **NGNGTSSEEV** **NRLKTR**IRNL KR**SLEICSKD** **ESELYSAYK**T
101 KLGR**AREQIS** **NLQESLRRER** **ESDKTDSYYR** **R**ELTR**ERNKI** **VELEKELNK**C
151 FDAKYIDEIN SKK**TRISDLE** **RQLAACK**SNG GSNGNMDQYK **REIESLKREL**
201 **AECRRGNNGS** **HSDCKYYDEE** **AR**DCVKS

Reference sequence: Q80HV0, 17 kDa A-type inclusion protein OS=Vaccinia virus (strain Western Reserve)
Protein sequence coverage: 59%

1 **METIKALEKF** **MEFDRLQK**DC SDKLDREKER RMKAER**EIAR** **KNCGGNPCER**
51 **ELESERSNVK** **RLEYQLDAEK** **EKVKFYKREL** **ERDRYLSSRY** **LTSSSDPHEK**
101 **PLPNYTFPR**I KNVSPLTTEA TGSVEVAPPS TDVTEPISDV TPSVDVEPEH
151 PPAF

Reference sequence: Q0SZY6, 50S ribosomal protein L2 OS=Shigella flexneri serotype 5b (strain 8401)
Protein sequence coverage: 73%

1 MAVVKCKPTS PGRR**HVVKVV** **NPELHKGKPF** **APLLEKNSK**S GGRNNNGRIT
51 TRHIGGGHKQ AYR**IVDFKRN** **KDGIPAVVER** **LEYDPNRSAN** **IALVLYKDGE**
101 **RRYILAPKGL** **KAGDQIQSGV** **DAAIKPGNTL** **PMRNIPVGST** **VHNVEMKPGK**
151 **GGQLARSAGT** **YVQIVARDGA** **YVTLRLR**SGE MRKVEADCR**A** **TLGEVGNAEH**
201 **MLR**VLGKAGA AR**WRGVRPTV** **RGTAMNPVDH** **PHGGGEGRNF** **GKHPVTPWGV**
251 **QTK**GKKTRSN K**RTDKFIVRR** RSK

Reference sequence: P07240, Envelope protein H3 OS=Vaccinia virus (strain Western Reserve)
Protein sequence coverage: 31%

1 MAAAKTPVIV VPVIDRLPSE TFPNVHEHIN DQKFDDVKDN EVMPEKRNVV
51 VVKDDPDHYK DYAFIQWTGG NIR**NDDKYTH** **FFSGFCNTMC** **TEETKR**NIAR
101 **HLALWDSNFF** **TELENKKVEY** **VVIVENDNVI** **EDITFLRPVL** **K**AMHDKKIDI
151 LQMREIITGN KVK**TELVMDK** **NHAIFTYTGG** **YDVSLSAYII** **R**VTTELNIVD
201 EIIKSGGLSS GFYFEIARIE NEMKINRQIL DNAAKYVEHD PRLVAEHR**FE**
251 **NMKPNFWSR**I GTAATKRYPG VMYAFTTPLI SFFGLFDINV IGLIVILFIM
301 FMLIFNVKSK LLWFLTGTFV TAFI

Reference sequence: P0DOT7, Protein L1 OS=Variola virus (isolate Human/India/Ind3/1967)
Protein sequence coverage: 38%

1 M**GAAASIQTT VNTLSER**ISS KLEQEANASA QTKCDIEIGN FYIRQNHGCN

51 LTVK**NMCSAD ADAQLDAVLS AATETYSGLT PEQKAYVPAM FTAALNIQTS**

101 **VNTVVR**DFEN YVKQTCNSSA VVDNKLK**IQN VIIDECYGAP GSPTNLEFIN**

151 **TGSSK**GNCAI KALMQLTTKA TTQIAPRQVA GTGVQFYMIV IGVIILAALF

201 MYYAKRMLFT STNDKIKLIL ANKENVHWTT YMDTFFRTSP MVIATTDIQN

Remarks/ Contamination: Slight contamination with other vaccinia virus proteins (possible cross-contamination during protein expression and purification), large number of other *E. coli* proteins and 50S ribosomal protein L2.

Protein: ATI-N-CPXV 1-725HIS

Reference sequence: P16602, A-type inclusion protein A25 homolog OS=Cowpox virus

Protein sequence coverage: 49%

1 MEVTNLIEK**C TKHSKDFATE VEKLWNDELS SESGLSRKTR NVIRNILRDI**

51 **TKSLTTDKK**S KCFR**ILERST INGEQIKDVY KTIFNNGVDV ESR**INTTGKY

101 VLFTVMTYAA AELR**LIKSDE IFALLSRFFN MICDIHRK**YG CGNMFVGIPA

151 ALINLLEIDQ INK**LFSVFST RYDAK**TFIYT EYFLFLNINH YLLVSGSELF

201 INVAYGPASF SSPISVPDYI MEALTFK**ACD HIMKSGDLKY TYAFTKKVKD**

251 **LFNTKSDSVY QYVRLHEMSY DGVSEDTDDD DEVFAILNLS IDSSVDR**YRN

301 **RVLLLTPEVA SLRKEYSDVE PDYKYLMDEE VPAYDKHLPK PITNTGIEEP**

351 **HATGGDK**EEQ EQQPVKVVQS KPDDGITPYN PFEDPDYVPT ITK**TVLGIAD**

401 **YQLVINKLIE WLDKCEEECG NGGEYKTELE EAKRKLTELN LELSDKLSKI**

451 **RTLERDSVYK TERIDRLTKE IK**ELR**DIQNG TDDGSDSSEI DKK**TIR**ELRE**

501 **SLDREREMRT ELERELDTIR DGKVEGSCQR ELELSRMWLK QRDDDLRAEI**

551 **DKRRNVEWEL SRLR**RDIKE**C DKYKEDLDKA KTTISNYVSR ISTLESEIAK**

601 **YQQDRDTLSV VRRELEEERR** RVRDLESRLD **ECTRNQEDTQ EVDALRSRIR**

651 **ELENKLADCM ESGGGNLTEI SR**LQSK**ISDL ER**QLRECRGN ATEISRLQYR

701 ITDLERQLND CRRNNENNAD TEREMQRLRD RITDLERQLS DCRRNNESNA

751 DMEREMQRLR DR**IMDLDR**QL NECKRDGNGT SSEEVNRLKT RIRDLER**SLE**

801 **ICSKDESELY SAYKSELGR**A R**EQISNLQES LRR**ERESDKT DSYYRRELTR

851 ERNKIVELEK ELNKCFDTNH AKYIDEINSK KTRISDLERQ LAACK**SNGGS**

901 **NGDMDQYKR**E IESLKRELAE CR**RGNNGSHS DCKYYDEEAR** EEVKR**LRQEL**

951 **TQLHEDLKR**A RESDKNDSYY KRELERQRAK VIEVEKELER YFDDSRLAEC

1001 KRHGDEMLRK IADLEKKLRD GGNGNGGNGC TSSCEFERKR IAVLEVEVRK

1051 SMETIKSLEK **FMEFDR**LQKD CADKLDREKE RR**MKAERDLE REIARKNCGG**

1101 **NPCER**ELESE RSNVKRLEYQ LDAEKEKVKF YKRELERDRY LSSRYLTSSS

1151 DPDEKPLPNY TFPRIEVEPL TTEDTEPKPV EVVPPSSDVT EPISSGVTPS

1201 VDAEPEHPQL SEYQTSVSQV AVTPPPKPET PQISEYQDYS ELYSASNNTE

1251 SKNVFSELAY LDDLDKLDDI DEYLLNNIMP EKTV

Remarks/ Contamination: Peptides after amino acid 725 due to carry-over from the column (very low signal intensity)

Reference sequence: P24759, A-type inclusion protein A25-VACV OS=Vaccinia virus (strain Western Reserve)

Protein sequence coverage: 51%

1 MEVTNLIEKC TKHSKDFATE VK**KLWNDELS** **SESGLSRKTR** **NVIRNILRDI**
51 **TKSLTTDKK**S KCFR**ILERST** **INGEQIKDVY** **KTIFNNGVDV** **ESR**INTTGKY
101 VLFTVMTYVA AELR**LIKSDE** **IFALLSRFFN** **MICDIHRK**YG CGNMFVGIPA
151 ALIILLEIDH INK**LFSVFST** **RYDAK**AYLYT EYFLFLNINH YLLSGSDLFI
201 NVAYGAVSFS SPISVPDYIM EALTFK**ACDH** **IMKSGDLKYT** **YAFTKKVKDL**
251 **FNTK**SDSIYQ YVR**LHEMSYD** **GVSEDTDDDD** **EVFAILNLSI** **DSSVDR**YR**NR**
301 **VLLLTPEVAS** **LRKEYSDVEP** **DYKYLMDEEV** **PAYDK**HLPKP ITNTGIEEPH
351 ATGGDEDQPI KVVHPPNNDK DDAIKPYNPL EDPNYVPTIT RTAIGIADYQ
401 LVINK**LIEWL** **DK**CEEECGNS GEFK**TELEEA** **KR**KLTELNAE LSDKLSK**IRT**
451 **LERDSVYKTE** **RIDRLTKEIK** EHRDIQNGTD DGSDLLEIDK KTIR**ELRESL**
501 **DRER**EMRSEL EK**ELDTIR**NG KVDGSCQR**EL** **ELSRMWLKQR** **DDDLRAEIDK**
551 **RRNVEWELSR** **LR**RDIKECDK **YKEDLDKAK**T TISNYVSK**IS** **TLESEIAKYQ**
601 **QDRDTLSVVR** **RELEEERR**RV **RDLESRLDEC** **TRNQEDTQEV** **DALRSRIREL**
651 **ENK**LTDCIES GGGNLTEISR LQSK**ISDLER** QLSECRENAT EISRLQSRIS
701 DLERQLNDCR RNNETNAETE RDATS

Remarks/ Contamination: Significant contamination with *E. coli* proteome.

Protein: B5-VACV

Reference sequence: Q01227, Protein B5-VACV OS=Vaccinia virus (strain Western Reserve)

Protein sequence coverage: 38%

1 MKTISVVTLL CVLPAVVYST CTVPTMNNAK **LTSTETSFND** **KQKVTFTCDQ**
51 **GYHSSDPNAV** **CETDKWKYEN** **PCKK**MCTVSD YISELYNKPL YEVNSTMTLS
101 CNGETKYFRC EEKNGNTSWN DTVTCPNAEC QPLQLEHGSC QPVKEKYSFG
151 EYMTINCDVG YEVIGASYIS CTANSWNVIP SCQQK**CDMPS** **LSNGLISGST**
201 **FSIGGVIHLS** **CKSGFTLTGS** **PSSTCIDGKW** **NPVLPICVRT** **NEEFDPVDDG**
251 **PDDETDLSKL** **SK**DVVQYEQE IESLEATYHI IIVALTIMGV IFLISVIVLV
301 CSCDKNNDQY KFHKLLP

Reference sequence: B5-VACV (provided by supplier)

Protein sequence coverage: 57%

1 **DLHHHHHHTC** **TVPTMNNAKL** **TSTETSFNDK** **QKVTFTCDQG** **YHSSDPNAVC**
51 **ETDKWKYENP** **CKK**MCTVSDY ISELYNKPLY EVNSTMTLSC NGETKYFRCE
101 EKNGNTSWND TVTCPNAECQ PLQLEHGSCQ PVKEKYSFGE YMTINCDVGY
151 EVIGASYISC TANSWNVIPS CQQK**CDMPSL** **SNGLISGSTF** **SIGGVIHLSC**
201 **KSGFTLTGSP** **SSTCIDGKWN** **PVLPICVRTN** **EEFDPVDDGP** **DDETDLSKLS**
251 **KDVVQYEQEI** **ESLE**

Remarks/ Contamination: Significant contamination with baculovirus proteins

Protein: B6-MPXV

Reference sequence: P24084, Protein B5- OS=Vaccinia virus (strain LC16m0)

Protein sequence coverage: 27%

1 MKTISVVTLL CVLPAVVYST CTVPTMNNAK **LTSTETSFND** **KQKVTFTCDQ**
51 **GYHSLDPNAV** **CETDKWKYEN** **PCKK**MCTVSD YVSELYDKPL YEVNSTMTLS

101 CNGETKYFRC EEKNGNTSWN DTVTCPNAEC QPLQLEHGSC QPVKEKYSFG
151 EYMTINCDVG YEVIGASYIS CTANSWNVIP SCQQKCDMPS LSNGLISGST
201 FSIGGVIHLS CK**SGFTLTGS** **PSSTCIDGKW** **NPILPTCVR**S NEK**FDPVDDG**
251 **PDDETDLSK**L SKDVVQYEQE IESLEATYHI IIVALTIMGV IFLISVIVLV
301 CSCDKNNDQY KFHKLLP

Reference sequence: B6-MPXV Thr20-His279 (provided by supplier)
Protein sequence coverage: 51%

1 MKTISVVTLL CVLPAVVYST CTVPTMNNAK **LTSTETSFND** **KQKVTFTCDS**
51 **GYHSLDPNAV** **CETDKWKYEN** **PCKK**MCTVSD YVSELYDKPL YEVNSTMTLS
101 CNGETKYFRC EEKNGNTSWN DTVTCPNAEC QPLQLEHGSC QPVK**EKYSFG**
151 **EYMTINCDVG** **YEVIGVSYIS** **CTANSWNVIP** **SCQQKCDIPS** **LSNGLISGST**
201 **FSIGGVIHLS** **CKSGFTLTGS** **PSSTCIDGKW** **NPILPTCVRS** **NEEFDPVDDG**
251 **PDDETDLSKL** **SK**DVVQYEQE IESLEATYHI IIMALTIMGV IFLISIIVLV
301 CSCDKNNDQY KFHKLLP

Remarks/ Contamination: Significant contamination with human proteome and rat proteins.

Protein: D8-VACV

Reference sequence: P04195, Cell surface-binding protein OS=Vaccinia virus (strain Western Reserve)
Protein sequence coverage: 67%

1 **MPQQLSPINI** **ETKKAISNAR** **LKPLDIHYNE** **SKPTTIQNTG** **KLVRINFKGG**
51 **YISGGFLPNE** **YVLSSLHIYW** **GKEDDYGSNH** **LIDVYKYSGE** **INLVHWNKKK**
101 **YSSYEEAKK**H DDGLIIISIF LQVLDHK**NVY** **FQKIVNQLDS** **IRSANTSAPF**
151 **DSVFYLDNLL** **PSK**LDYFTYL GTTINHSADA VWIIFPTPIN IHSDQLSK**FR**
201 **TLLSSSNHDG** **KPHYITENYR** **NPYKLNDDTQ** **VYYSGEIIRA** **ATTSPARENY**
251 **FMRWLSDLR**E TCFSYYQKYI EENKTFAIIA IVFVFILTAI LFFMSRRYSR
301 EKQN

Reference sequence: P0DOT7, Protein L1-VACV OS=Variola virus (isolate Human/India/Ind3/1967)
Protein sequence coverage: 54%

1 M**GAAASIQTT** **VNTLSERISS** **KLEQEANASA** **QTKCDIEIGN** **FYIR**QNHGCN
51 LTVK**NMCSAD** **ADAQLDAVLS** **AATETYSGLT** **PEQKAYVPAM** **FTAALNIQTS**
101 **VNTVVRDFEN** **YVK**QTCNSSA VVDNKLK**IQN** **VIIDECYGAP** **GSPTNLEFIN**
151 **TGSSKGNCAI** **K**ALMQLTTKA TTQIAPRQVA GTGVQFYMIV IGVIILAALF
201 MYYAKRMLFT STNDKIKLIL ANKENVHWTT YMDTFFRTSP MVIATTDIQN

Reference sequence: D8-VACV 2-260HIS (provided by supplier)
Protein sequence coverage: 66%

1 **MPQQLSPINI** **ETKKAISNAR** **LKPLDIHYNE** **SKPTTIQNTG** **KLVRINFKGG**
51 **YISGGFLPNE** **YVLSSLHIYW** **GKEDDYGSNH** **LIDVYKYSGE** **INLVHWNKKK**
101 **YSSYEEAKK**H DDGLIIISIF LQVSDHK**NVY** **FQKIVNQLDS** **IR**SANTSAPF
151 DSVFYLDNLL PSTLDYFTYL GTTIK**HSADA** **VWIIFPTPIN** **IHSDQLSK**FR
201 **TLLSSSNHDG** **KPYYITENYR** NPYK**LNDDTQ** **VYYSGEIIRA** **ATTSPARENY**
251 **FMRWLSDLR**E TCFSYYQKYI EGNKTFAIIA IVFVFILTAI LFLMSRRYSR
301 EKQN

Remarks/ Contamination: Significant contamination with *E. coli* proteome.

Protein: E8-MPXVL
Reference sequence: Q8V4Y0, Cell surface-binding protein OS=Monkeypox virus (strain Zaire-96-I-16)
Protein sequence coverage: 70%

1 MPQQLSPINI ETKKAISDTR LKTLDIHYNE SKPTTIQNTG KLVRINFKGG
51 YISGGFLPNE YVLSTIHIYW GKEDDYGSNH LIDVYKYSGE INLVHWNKKK
101 YSSYEEAKKH DDGIIIIAIF LQVSDHKNVY FQKIVNQLDS IRSANMSAPF
151 DSVFYLDNLL PSTLDYFTYL GTTINHSADA AWIIFPTPIN IHSDQLSKFR
201 TLLSSSNHEG KPHYITENYR NPYKLNDDTQ VYYSGEIIRA ATTSPVRENY
251 FMKWLSDLRE ACFSYYQKYI EGNKTFAIIA IVFVFILTAI LFLMSQRYSR
301 EKQN

Reference sequence: P68616, Protein A33-VACV OS=Vaccinia virus (strain Copenhagen)
Protein sequence coverage: 29%

1 MMTPENDEEQ TSVFSATVYG DKIQGKNKRK RVIGLCIRIS MVISLLSMIT
51 MSAFLIVRLN QCMSANEAAI TDAAVAVAAA SSTHRKVASS TTQYDHKESC
101 NGLYYQGSCY ILHSDYQLFS DAKANCTAES STLPNKSDVL ITWLIDYVED
151 TWGSDGNPIT KTTSDYQDSD VSQEVRKYFC VKTMN

Protein sequence: E8-MPXV_Pro2-Lys274 (provided by supplier)
Protein sequence coverage: 70%

1 MPQQLSPINI ETKKAISDTR LKTLDIHYNE SKPTTIQNTG KLVRINFKGG
51 YISGGFLPNE YVLSTIHIYW GKEDDYGSNH LIDVYKYSGE INLVHWNKKK
101 YSSYEEAKKH DDGIIIIAIF LQVSDHKNVY FQKIVNQLDS IRSANMSAPF
151 DSVFYLDNLL PSTLDYFTYL GTTINHSADA AWIIFPTPIN IHSDQLSKFR
201 TLLSSSNHEG KPHYITENYR NPYKLNDDTQ VYYSGEIIRA ATTSPVRENY
251 FMKWLSDLRE ACFSYYQKYI EGNKTFAIIA IVFVFILTAI LFLMSQRYSR
301 EKQN

Remarks/ Contamination: Significant contamination with human proteome.

Protein: H3-VACV
Reference sequence: P07240, Envelope protein H3-VACV OS=Vaccinia virus (strain Western Reserve)
Protein sequence coverage: 66%

1 MAAAKTPVIV VPVIDRLPSE TFPNVHEHIN DQKFDDVKDN EVMPEKRNVV
51 VVKDDPDHYK DYAFIQWTGG NIRNDDKYTH FFSGFCNTMC TEETKRNIAR
101 HLALWDSNFF TELENKKVEY VVIVENDNVI EDITFLRPVL KAMHDKKIDI
151 LQMREIITGN KVKTELVMDK NHAIFTYTGG YDVSLSAYII RVTTELNIVD
201 EIIKSGGLSS GFYFEIARIE NEMKINRQIL DNAAKYVEHD PRLVAEHRFE
251 NMKPNFWSRI GTAATKRYPG VMYAFTTPLI SFFGLFDINV IGLIVILFIM
301 FMLIFNVKSK LLWFLTGTFV TAFI

Reference sequence: P0DOT7, Protein L1-VACV OS=Variola virus (isolate Human/India/Ind3/1967)
Protein sequence coverage: 58%

1 MGAAASIQTT VNTLSERISS KLEQEANASA QTKCDIEIGN FYIRQNHGCN
51 LTVKNMCSAD ADAQLDAVLS AATETYSGLT PEQKAYVPAM FTAALNIQTS

101 **VNTVVR**DFEN YVKQTCNSSA VVDNK**LKIQN VIIDECYGAP GSPTNLEFIN**
151 **TGSSKGNCAI KALMQLTTKA TTQIAPR**QVA GTGVQFYMIV IGVIILAALF
201 MYYAKRMLFT STNDKIKLIL ANKENVHWTT YMDTFFRTSP MVIATTDIQN

Reference sequence: H3-VACV 21-270HIS (provided by supplier)
Protein sequence coverage: 62%

1 MAAVKTPVIV VPVIDRPPSE TFPNVHEHIN DQK**FDDVKDN EVMPEKRNVV**
51 **VVKDDPDHYK DYAFIQWTGG NIRNDDKYTH FFSGFCNTMC TEETKR**NIAR
101 **HLALWDSNFF TELENKKVEY VVIVENDNVI EDITFLRPVL KAMHDKKIDI**
151 **LQMREIITGN KVKTELVMDK** NHTIFTYTGG YDVSLSAYII **RVTTALNIVD**
201 **EIIKSGGLSS GFYFEIARIE NEMKINRQIL DNAAKYVEHD PRLVAEHRFE**
251 **NMKPNFWSR**I GTAAAKRYPG VMYAFTTPLI SFFGLFDINV IGLIVILFIM
301 FMLIFNVKSK LLWFLTGTFV TAFI

Remarks/ Contamination: Contamination with *E. coli* proteome.

Protein: L1-VACV
Reference sequence: P07612, Protein L1-VACV OS=Vaccinia virus (strain Western Reserve)
Protein sequence coverage: 70%

1 **MGAAASIQTT VNTLSERISS KLEQEANASA QTKCDIEIGN FYIRQNHGCN**
51 **LTVKNMCSAD ADAQLDAVLS AATETYSGLT PEQKAYVPAM FTAALNIQTS**
101 **VNTVVRDFEN YVKQTCNSSA VVDNKLKIQN VIIDECYGAP GSPTNLEFIN**
151 **TGSSKGNCAI KALMQLTTKA TTQIAPK**QVA GTGVQFYMIV IGVIILAALF
201 MYYAKRMLFT STNDKIKLIL ANKENVHWTT YMDTFFRTSP MVIATTDMQN

Reference sequence: L1-VACV (provided by supplier)
Protein sequence coverage: 92%

1 **DPAMGAAASI QTTVNTLSER ISSKLEQEAN ASAQTKCDIE IGNFYIRQNH**
51 **GCNLTVKNMC SADADAQLDA VLSAATETYS GLTPEQKAYV PAMFTAALNI**
101 **QTSVNTVVRD FENYVKQTCN SSAVVDNKLK IQNVIIDECY GAPGSPTNLE**
151 **FINTGSSKGN CAIKALMQLT TKATTQIAPK** QVAGTGVQHH HHHH

Remarks/ Contamination: High purity.

Protein: M1-MPXV
Reference sequence: P0DOT7, Protein L1-VACV OS=Variola virus (isolate Human/India/Ind3/1967)
Protein sequence coverage: 70%

1 M**GAAASIQTT VNTLSERISS KLEQEANASA QTKCDIEIGN FYIRQNHGCN**
51 **LTVKNMCSAD ADAQLDAVLS AATETYSGLT PEQKAYVPAM FTAALNIQTS**
101 **VNTVVRDFEN YVKQTCNSSA VVDNKLKIQN VIIDECYGAP GSPTNLEFIN**
151 **TGSSKGNCAI KALMQLTTKA TTQIAPR**QVA GTGVQFYMIV IGVIILAALF
201 MYYAKRMLFT STNDKIKLIL ANKENVHWTT YMDTFFRTSP MVIATTDIQN

Reference sequence: M1-MPXV Gly2-Gly183 (provided by supplier)
Protein sequence coverage: 70%

1 M**GAAASIQTT VNTLSERISS KLEQEANASA QTKCDIEIGN FYIRQNHGCN**

51 **ITVKNMCSAD ADAQLDAVLS AATETYSGLT PEQKAYVPAM FTAALNIQTS**
101 **VNTVVRDFEN YVKQTCNSSA VVDNKLKIQN VIIDECYGAP GSPTNLEFIN**
151 **TGSSKGNCAI KALMQLTTKA TTQIAPR**QVA GTGVQFYMIV IGVIILAALF
201 MYYAKRMLFT STNDKIKLIL ANKENVHWTT YMDTFFRTSP MIIATTDMQN

Remarks/ Contamination: Significant contamination with human proteome and rat proteins.

Bead-based multiplex assay

The multiplex bead mix containing 1000 beads per region for each antigen tested was freshly prepared for each measurement. 50 µL of the mix was added per well of a polystyrene 96 well flat bottom microplate (Greiner Bio-One, Frickenhausen, Germany) to which 50 µL of diluted serum or standard were added per well, then incubated for 1 hour at room temperature on a plate shaker (IKA MTS 2/4 digital, IKA-Werke, Staufen im Breisgau, Germany) at 750 rpm. Using a magnetic plate washer (HydroSpeed™, Tecan, Männedorf, Switzerland), plates were washed 3-times with 200 µL PBS/T (PBS + 0.1 % Tween 20) per well, using soak times of 1 minute for each washing cycle. Subsequently, 100 µL PE-labelled goat anti-human IgG antibody was added at a concentration of 1 µg/mL in PBS/B (PBS + 1% BSA) assay buffer to each well in the first six columns of each 96 well plate, and 100 µL PE-labelled donkey anti-human IgM antibody was added to the second six columns. Plates were incubated and washed as described before. Thereafter the washed beads were resuspended in 100 µL sheath fluid (PBS) per well by shaking for 1 min at 750 rpm on the plate shaker. Finally, fluorescent signals were analyzed on a Bio-Plex 200 instrument (Bio-Rad, Munich, Germany) using normal detector gain (RP1 target) collecting data from at least 50 beads per region. Raw data was exported into a Microsoft Excel® spreadsheet and further processed and analysed as described below.

Test of coupling efficiency with monospecific poly- or monoclonal antibodies

To test the coupling efficiency for the different orthopoxvirus-specific antigens, polyclonal, but monospecific antibodies directed against a single protein (anti D8-VACV, anti H3-VACV, anti A33-VACV, and anti L1-VACV) were biotinylated using Biotinamidohexanoyl-6-aminohexanoic acid-N-hydroxysuccinimide ester (Sigma-Aldrich) at a molar coupling ratio of 20. For the detection of A27-VACV monoclonal antibody A1/40 and for B5-VACV monoclonal antibody NR-429 were used. Polyclonal antibodies were tested at final concentrations of 10, 2, and 0.4 µg/mL, monoclonal antibodies at final concentrations of 1, 0.2 and 0.04 µg/mL while VIG was tested at 1:100, 1:500 and 1:2500 dilutions diluted in assay buffer (PBS, 1% BSA) as described above. Biotinylated polyclonal antibodies were detected using SA-PE at 2 µg/mL, mouse monoclonal antibodies were detected using goat anti-mouse IgG (Fc-γ specific) PE-labelled antibodies, and VIG was detected using goat anti-human IgG (Fc-γ specific) PE-labelled antibodies at a final concentration of 1 µg/mL each. Batch-to-batch variability for each new batch of coupled beads was tested as described with a 1:4 dilution series of VIG starting at a 1:100 dilution in a total of 7 dilutions together with a buffer only blank control in technical duplicates.

Supplementary references

1 Stern, D. *et al.* Development of a Genus-Specific Antigen Capture ELISA for Orthopoxviruses - Target Selection and Optimized Screening. *PLoS One* **11**, e0150110 (2016). https://doi.org/10.1371/journal.pone.0150110

2 Kurth, A. *et al.* Rat-to-elephant-to-human transmission of cowpox virus. *Emerg Infect Dis* **14**, 670-671 (2008). https://doi.org/10.3201/eid1404.070817

3 Worbs, S. *et al.* Differentiation, Quantification and Identification of Abrin and Abrus precatorius Agglutinin. *Toxins (Basel)* **13** (2021). https://doi.org/10.3390/toxins13040284

4 UniProt, C. UniProt: the Universal Protein Knowledgebase in 2023. *Nucleic Acids Res* **51**, D523-D531 (2023). https://doi.org/10.1093/nar/gkac1052
